# Environmental controls on the light use efficiency of terrestrial gross primary production

**DOI:** 10.1111/gcb.16511

**Published:** 2022-11-25

**Authors:** Keith J. Bloomfield, Benjamin D. Stocker, Trevor F. Keenan, I. Colin Prentice

**Affiliations:** ^1^ Georgina Mace Centre for the Living Planet, Department of Life Sciences, Imperial College London Ascot UK; ^2^ Department of Environmental Systems Science, ETH Zurich Switzerland; ^3^ Swiss Federal Institute for Forest, Snow and Landscape Research WSL Birmensdorf Switzerland; ^4^ Institute of Geography University of Bern Bern Switzerland; ^5^ Oeschger Centre for Climate Change Research University of Bern Bern Switzerland; ^6^ Department of Environmental Science, Policy and Management, UC Berkeley Berkeley California USA; ^7^ Climate and Ecosystem Sciences Division, Lawrence Berkeley National Laboratory Berkeley California USA; ^8^ Department of Biological Sciences Macquarie University North Ryde New South Wales Australia; ^9^ Ministry of Education Key Laboratory for Earth System Modelling, Department of Earth System Science Tsinghua University Beijing China

**Keywords:** diffuse radiation, eddy covariance, FLUXNET, light use efficiency, soil moisture, temperature, terrestrial biosphere model, vapor pressure deficit

## Abstract

Gross primary production (GPP) by terrestrial ecosystems is a key quantity in the global carbon cycle. The instantaneous controls of leaf‐level photosynthesis are well established, but there is still no consensus on the mechanisms by which canopy‐level GPP depends on spatial and temporal variation in the environment. The standard model of photosynthesis provides a robust mechanistic representation for C_3_ species; however, additional assumptions are required to “scale up” from leaf to canopy. As a consequence, competing models make inconsistent predictions about how GPP will respond to continuing environmental change. This problem is addressed here by means of an empirical analysis of the light use efficiency (LUE) of GPP inferred from eddy covariance carbon dioxide flux measurements, in situ measurements of photosynthetically active radiation (PAR), and remotely sensed estimates of the fraction of PAR (fAPAR) absorbed by the vegetation canopy. Focusing on LUE allows potential drivers of GPP to be separated from its overriding dependence on light. GPP data from over 100 sites, collated over 20 years and located in a range of biomes and climate zones, were extracted from the FLUXNET2015 database and combined with remotely sensed fAPAR data to estimate daily LUE. Daytime air temperature, vapor pressure deficit, diffuse fraction of solar radiation, and soil moisture were shown to be salient predictors of LUE in a generalized linear mixed‐effects model. The same model design was fitted to site‐based LUE estimates generated by 16 terrestrial ecosystem models. The published models showed wide variation in the shape, the strength, and even the sign of the environmental effects on modeled LUE. These findings highlight important model deficiencies and suggest a need to progress beyond simple “goodness of fit” comparisons of inferred and predicted carbon fluxes toward an approach focused on the functional responses of the underlying dependencies.

## Graphical abstract

A common dataset of environmental variables, including temperature and measures of air and soil moisture, produced widely differing effects when applied to productivity predictions generated by an ensemble of terrestrial biosphere models. The published models showed variation in the shape, the strength, and even the sign of the environmental effects on modeled light use efficiency (mol C mol^−1^ photons).

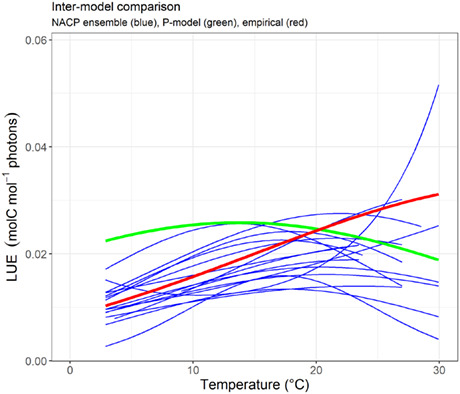

## 1 INTRODUCTION

Gross primary production (GPP) by terrestrial ecosystems is the largest flux in the global carbon cycle, and responsible for the annual uptake of approximately 120–150 Pg carbon: about one‐sixth of the total amount of carbon dioxide (CO_2_) in the atmosphere (Beer et al., 2010). GPP, expressed over a period, represents the integrated, canopy‐level response of photosynthesis to climate, nutrient availability, and disturbance (Law et al., 2002). Accurate estimation of GPP, including its geographic pattern and its temporal variability and trends, is essential if we are to understand the full implications of the anthropogenic increase of atmospheric CO_2_ and the associated changes in climate.

Today's Earth system models, global models that represent coupled physical and biogeochemical processes, are unprecedented in scope and scale (Fisher & Koven, 2020). Thanks to technological advances that allow continuous monitoring and remote sensing of land ecosystems, as well as continuous improvement in computational resources, we should now be able to profit from advanced research tools and a huge body of data that were unavailable to earlier researchers (Eyring et al., 2021). Perhaps surprisingly, however, fundamental questions about how terrestrial ecosystems function remain unanswered. The nature of the responses of primary production to continuing changes in CO_2_ and climate is among the most critical (Walker et al., 2021), as it influences every aspect of environmental change impacts on terrestrial biological systems.

The challenge of modeling ecosystem dynamics globally has fueled the development of an increasing number of competing terrestrial biosphere models (TBMs; e.g., Fisher et al., 2014). This proliferation has led to a wide spread of predictions (e.g., Arora et al., 2020; Friedlingstein et al., 2006), both by TBMs and by Earth system models in which they are embedded. The complexity of TBMs has generally increased over time, but this has not improved their accuracy in predicting GPP (Prentice et al., 2015). Persistent disparities between models have propelled a movement toward standardized model benchmarking and evaluation (Collier et al., 2018; Kelley et al., 2013), but models still show no sign of convergence. The fundamental problem is that whereas the instantaneous controls of leaf‐level photosynthesis (which can be measured in manipulative experiments) are well established, the longer term, larger scale controls of GPP are not. For example, in the standard model of C_3_ photosynthesis (Farquhar von Caemmerer Berry [FvCB], Farquhar et al., 1980), carbon assimilation is accurately modeled as the lesser of two rates, limited either by Rubisco activity (carboxylation) or by light (electron transport). Both rates are influenced by the leaf‐internal partial pressure of CO_2_. Empirical “closure” equations are commonly used to determine this quantity, which depends on photosynthetic rate and stomatal conductance as well as on ambient CO_2_. Additional assumptions are needed, however, about how key parameters of the FvCB model (which can be measured directly on individual leaves, but not on whole canopies) and parameters of the closure equations should be scaled up in space and time (e.g., Rogers et al., 2017). These assumptions are seldom tested, or even made explicit in published model applications. Yet they induce major differences in how modeled GPP responds to light, temperature, CO_2_, nutrient availability, and other aspects of the physical environment (Friedlingstein et al., 2014).

Light use efficiency (LUE) is a simplifying concept exploited here to analyze environmental controls of GPP, and to compare these between models and data. The LUE principle was first established in studies of crop growth by Monteith (1972, 1977). It states that primary production over a given period (of a week or longer) is proportional to the light absorbed by the canopy during that period—which, in turn, is the product of the incident photosynthetic photon flux density (PPFD) and the fractional absorbed photosynthetically active radiation (fAPAR):

(1) GPP=PPFD×fAPAR×LUE.

This equation provides a basis for remote sensing of GPP, using spectral reflectance measurements that can be used to estimate fAPAR (e.g., Gobron et al., 2003). Numerous algorithms are used to estimate LUE and many of these depend on land cover classifications and on environmental variables including temperature, vapor pressure deficit (VPD), and (occasionally) CO_2_. However, almost all satellite‐based models for GPP are based, in one way or another, on Equation (1). Although the strongly nonlinear relationship between instantaneous leaf photosynthesis and absorbed light is well understood (and captured within the FvCB model), a different relationship emerges for plant canopies at timescales of a week or longer—those periods originally considered by Monteith. Investigating the physiological basis underpinning the LUE model, Medlyn (1998) found support for the proposal that variability in LUE is reduced, and its relationship with light becomes more linear, as the time‐step considered increases. We return to this point in the Discussion.

The LUE term in Equation (1) represents the outcome of all photosynthetic processes including Rubisco kinetics and acclimation (Scafaro et al., 2017; Togashi et al., 2018), electron transport, and stomatal behavior. Focusing on LUE (rearranging Equation 1) permits us to analyze potential drivers of GPP independent of its overriding control by absorbed light. LUE is found to be relatively conservative during the growing season and when water is non‐limiting (e.g., Barr et al., 2007), but is not necessarily fixed even for mature forests (Urbanski et al., 2007). fAPAR is both a key control of GPP and ultimately derived from it through the allocation of carbon to leaf construction and the relationship between leaf area index (LAI) and light absorbance. Most TBMs simulate LAI, and its seasonal variation, as well as LUE—albeit with, so far, limited success (Kelley et al., 2013; Park & Jeong, 2021). For canopies, the diffuse fraction of the incident radiation is also important and eddy covariance studies have demonstrated that productivity is greater on overcast than on clear days (e.g., Hollinger et al., 1994). The volume of shade within a canopy is lower under cloudy compared to full beam conditions (Roderick et al., 2001) and so penetration of available light into the canopy profile is increased such that a greater proportion reaches lower leaves relative to leaves at the canopy surface. The effect on canopy productivity entrained by the reduction in radiation load on sunlit leaves is minor while the photosynthesis of shade leaves has an essentially linear response to radiation since they are seldom light‐saturated (dePury & Farquhar, 1997). The effect on productivity of deeper penetration by diffuse radiation is most pronounced in dense canopies (Knohl & Baldocchi, 2008) and so we can hypothesize an interaction between diffuse fraction and some measure of vegetation amount (Wang et al., 2018).

The eddy covariance method provides measurements of net carbon fluxes between the canopy and atmosphere (net ecosystem exchange, NEE) over a range of timescales from half‐hourly to multi‐annual (Baldocchi, 2020). Those measurements, when partitioned into GPP and ecosystem respiration (*R*_eco_), allow empirical evaluation of GPP estimates generated by TBMs. FLUXNET, an international network now offering standardized variables at over 200 sites, has been operating for more than two decades (Pastorello et al., 2020). Key climate variables including radiation components, VPD, and air temperature are captured by the micrometeorological instruments that record data alongside the carbon flux measurements. Vegetation cover and soil moisture, however, are not routinely captured in FLUXNET. The scientific value of the network continues to grow as the length of individual site records increases. Many flux‐tower sites established in the mid‐1990s now provide datasets capable of capturing decadal trends, which can be analyzed statistically (Fernández‐Martínez et al., 2017) and compared with model simulations (e.g., Urbanski et al., 2007). Exploiting the global distribution and temporal span of FLUXNET data, here we (a) use open‐source FLUXNET data on GPP and environmental variables, together with remotely sensed fAPAR data, to provide a parsimonious empirical model for LUE; (b) interpret the resulting model in terms of the functional forms defining the relationships between LUE and climate; and (c) compare those functional forms against corresponding relationships derived from a published set of TBM outputs.

## 2 METHODS

### 2.1 Data

We used the FLUXNET2015 Tier 1 dataset of daily eddy covariance data restricted to those values for which less than half of the underlying half‐hourly data were gap‐filled. Following Stocker et al. (2018), we excluded those sites classified as croplands, or wetlands and sites where C_4_ vegetation was either mentioned in the site description, or expected to dominate. Our final dataset (after further filtering described below) retained 117 sites covering nine vegetation types (Figure S1; Table S1). Vegetation categories follow the IGBP land cover classification system: evergreen needleleaf forest (ENF), evergreen broadleaf forest (EBF), deciduous broadleaf forest (DBF), mixed forest (MF), closed shrublands (CSH), open shrublands (OSH), woody savannahs (WSA), savannahs (SAV), and grasslands (GRA). Daily GPP data used were based on the nighttime flux decomposition method and the filtering based on a variable friction velocity threshold (GPP_NT_VUT_REF). PPFD (mol photons m^−2^ day^−1^) was estimated as a constant fraction of downwelling, shortwave radiation (SW_IN_F, W m^−2^) using a conversion factor of 2.04 μmol J^−1^ (Meek et al., 1984). VPD (VPD_F, Pa) and CO_2_ (CO2_F_MDS, μmol mol^−1^) data were restricted to daytime conditions by averaging data from half‐hourly time‐steps with positive insolation (SW_IN_F). Air temperature data are given directly (TA_F_DAY).

Diffuse radiation data (*S*_d_; converted as above from variable PPFD_DIF, μmol m^−2^ s^−1^) were only available for 31 sites and were often incomplete. As an alternative variable, we calculated a Cloudiness Index based on observed and potential solar radiation (e.g., Turner, Ritts, Styles, et al., 2006; Wang et al., 2018):

(2) $$\mathrm{CI}=1-\left(S_{t}/S_{o}\right),$$

where *S*_t_ is short‐wave radiation at the surface (SW_IN_F) and *S*_*o*_ is potential radiation at the top of the atmosphere (variable SW_IN_POT). In a second approach, we plotted the available diffuse transmittance (*T*_*d*_ = *S*_*d*_/*S*_*o*_) fractions against corresponding values for total transmittance (*T*_*T*_ = *S*_*t*_/*S*_*o*_) to fit a predictive equation as given by Bristow et al. (1985):

(3) $$T_{d}=T_{T}\;\left[1-e^{\left\{0.6∙\left(\left(1-B/T_{T}\right)/\left(B-0.4\right)\right)\right\}}\right].$$

The empirical fit of Equation (3) yielded a *B* coefficient of 0.889 (Figure S2) and was used to gap‐fill the *T*_*d*_ estimates.

Values of fAPAR at the FLUXNET sites were obtained using two remotely sensed products: MODIS FPAR (MCD15A3H, at a resolution of 500 m and 4 days) and MODIS EVI (MOD13Q1, at a resolution of 250 m and 8 days). Data were downloaded for the pixel in which each tower is located from the *google_earth_engine_subsets* library (Hufkens, 2017). Data were then filtered to remove contamination associated with clouds, values of unity, and likely outliers (more than three times the inter‐quartile range). Filtered values were then interpolated to daily estimates using a cubic smoothing spline. Time‐series plots were reviewed for reasonableness; none of the interpolated values induced radical fluctuations in the underlying trend (sample FPAR plots are shown at Figure S3). Reasonable agreement was found between the two indices (Figure S4). We adopted FPAR for the main analysis in the expectation that it offers a more direct link to photosynthetic processes.

Soil moisture data are not routinely or uniformly available within FLUXNET (only 14 of the selected sites report soil water at depths >50 cm) and so we rely here on model‐based estimates (Stocker et al., 2020) provided by a version of the SPLASH model (Davis et al., 2017) extended to allow variation in water‐holding capacity based on soil texture and depth data up to a maximum of 2 m, extracted for site locations from SoilGrids data (Hengl et al., 2014).

### 2.2 Data processing and exploration

The site‐day dataset was filtered to exclude negative GPP estimates, near‐zero absorbed light, and apparent LUE values deemed infeasible (>0.12 mol C mol^−1^ photons). The time‐series data were restricted to the growing season, defined by a simple threshold approach adapted from Lasslop et al. (2012). Following their approach, for a given site‐year, daily GPP data were scaled such that the 0.05 quantile was zero and the 0.95 quantile was equal to one; GPP values were retained if they exceeded 0.2 (i.e., 20% of the 0.05–0.95 quantile range).

LUE was calculated as the ratio of 15‐day accumulated GPP to the product of 15‐day accumulated photosynthetically active radiation (PAR) and the 15‐day average fAPAR. We adopted 15‐day non‐overlapping windows (e.g., Reichstein et al., 2005) and excluded stub periods with <15 consecutive days. Alternative timescales were appraised (daily and weekly; Figure S5), and the possible implications of choice of time‐step are considered later. The final dataset (growing season, 15‐day composites) contained 8049 rows.

### 2.3 Data‐model comparisons

GPP simulations were generated using an LUE model based on optimality principles (P‐model, Wang et al., 2017), extended to include temperature sensitivity of the intrinsic quantum yield and an empirical soil moisture stress function (implemented via the R package rpmodel, Stocker, 2019; Stocker et al., 2020). Briefly, the P‐model coordinates capacities for CO_2_ fixation, water‐ and electron transport to simulate GPP consistent with the FvCB framework; the model can be applied universally to C_3_ plants without the need for biome‐ or vegetation‐specific parameters. Matching site‐day GPP predictions were generated by forcing the P‐model with the combined FLUXNET_SPLASH dataset outlined above. Model simulations thus obtained were used to provide a direct comparison with our analysis of the FLUXNET data. We also carried out stylized experiments with the P‐model, varying one environmental driver at a time while holding the others constant (at median value), to test whether our statistical analysis of P‐model outputs correctly reproduced the environmental responses of the model's formulation.

The North American Carbon Program (NACP) has provided standardized output, including GPP and LAI, from 24 TBMs for 47 eddy covariance flux tower sites in North America (Ricciuto et al., 2013; Schaefer et al., 2012). Not all TBMs, however, provided GPP estimates for every site or time point. The NACP forcing data derive in part from sites that also form part of FLUXNET (Table S1). That overlap (12 sites within FLUXNET & NACP) allowed us to use common site‐based flux data to compare the environmental dependencies of LUE estimates predicted by our own empirical model, by the P‐model, and by 16 TBMs (Table S2). For the NACP comparisons, fAPAR estimates were calculated from the LAI simulations recognizing that light interception approximates to an exponential function of leaf area (Turner, Ritts, Cohen, et al., 2006):

(4) $$\text{fAPAR}≅1-e^{\left(-k\times \mathrm{LAI}\right)}.$$

We have assumed an extinction coefficient (*k*) of 0.5, a commonly used value, since even distal leaves on sun‐exposed branches only experience a fraction of full PAR incident at the horizontal plane (e.g., Kitajima et al., 2005).

### 2.4 Statistical analysis

The response variable in the analysis is LUE, defined as GPP normalized by absorbed light. Model selection, aimed at finding that design explaining the most variation in LUE with the minimum necessary parameters, was guided by Akaike and Bayesian information criteria (AIC and BIC, respectively; the lower the better in both cases), which provide objective measures of model performance by quantifying the trade‐off between explanatory power and complexity. A generalized model design that accommodates a non‐normal response distribution was strongly preferred (AIC were much lower for equivalent models adopting a gamma rather than a Gaussian distribution); that preference derives from the right‐skewed and zero‐truncated distribution of the inferred LUE values. Next, we considered a variance structure (random intercept term) that best reflected the hierarchical and longitudinal nature of the dataset and that recognized the lack of independence created by repeat measurements at a given site (AIC for year nested in site < site only < year only). The candidate predictors considered are daytime temperature and VPD, soil moisture, elevation above sea level, ambient CO_2_, and diffuse radiation—the last represented by an interaction between Cloudiness Index (Equation 2) and fAPAR. Transformations of the explanatory variables were considered, consistent with well‐documented, nonlinear response functions such as the classic humped photosynthetic temperature response (e.g., Berry & Björkman, 1980) and the exponential decrease in stomatal conductance with increasing VPD (e.g., Oren et al., 1999). Backward stepped selection (from more to less complex) of multiple predictor variables is presented in Table 1. The complexity of the starting model (M01, Table 1) generated convergence warnings suggesting potential numerical estimation constraints or overfitting, those difficulties were removed as the selection steps led to progressively simpler models.

**TABLE 1:** Model selection steps. The response variable is light use efficiency (LUE) and all models shared a common random design as per Equation (5). Candidate terms: daytime temperature (Temp), daytime vapour pressure deficit (VPD), soil moisture (Sm), elevation (Elv), ambient CO_2_, cloudiness index (CI), diffuse transmittance (*T*_d_) and fAPAR

Model	Fixed term	Rationale	df	AIC	BIC	logLik
M01	poly(Temp, 2) + log(VPD) + log(Sm) + Elv + CO_2_ + CI:fAPAR	Beyond optimal	11	−63,825	−63,749	31,924
M02	poly(Temp, 2) + log(VPD) + log(Sm) + Elv + CI:fAPAR	Drop CO_2_	10	−63,826	−63,756	31,923
M03	poly(Temp, 2) + log(VPD) + log(Sm) + CI:fAPAR	Drop elevation	9	−63,824	−63,761	31,921
M04	poly(Temp, 2) + log(VPD) + log(Sm)	Drop diffuse fraction	8	−63,758	−63,702	31,887
M05	poly(Temp, 2) + log(VPD) + log(Sm) + *T*_d_:fAPAR	Replace CI with *T*_diffuse_	9	−63,781	−63,718	31,899
M06	poly(Temp, 2) + log(VPD) + log(Sm) + CI	Drop fAPAR interaction	9	−64,464	−64,401	32,241
M07	poly(Temp, 2) + log(VPD) + CI	Drop soil moisture	8	−64,323	−64,267	32,170
M08	Temp + VPD + Sm + CI	Remove transformations	8	−64,371	−64,315	32,193

The shading served to highlight the preferred model that is then pursued in the subsequent analysis.

Abbreviations: AIC, Akaike Information Criterion; BIC, Bayesian Information Criterion; df, degrees of freedom; logLik, log likelihood.

Conditional plots, showing partial residuals, were employed to visualize the relationship between the fitted LUE response and each explanatory term while holding all other variables in the final model constant (Breheny & Burchett, 2017). For the conditioning, the other model variables are set to their median for numeric variables and to the most common level for categorical variables (here site and year).

## 3 RESULTS

### 3.1 Model evaluation

The starting, or maximal, model considered (M01, Table 1) was a generalized linear mixed‐effects model adopting a gamma distribution for LUE, with a random term recognizing the hierarchical structure of the data (repeat measurements at individual sites), and a fixed term comprising daytime temperature (as a second‐degree polynomial function), VPD, soil moisture (both log‐transformed), elevation, CO_2_, and a term providing for the interaction between diffuse radiation and fAPAR (CI: fAPAR). The final preferred model (M06, providing the lowest AIC per Table 1) was reached after successively dropping CO_2_, elevation, and the fAPAR interaction. Elimination of soil moisture (M07), however, produced a weaker model (higher AIC etc.) as did substitution of diffuse transmittance (*T*_d_) for Cloudiness Index (M05). Adoption of a basic model formulation (M08), with no transformations and with the temperature effect reduced to a single (linear) term, produced a much weaker outcome. The preferred model (M06, Table 1) is:

(5) $$\begin{array}{c} \log\left({\mathrm{LUE}}_{\mathrm{ijk}}\right)=\alpha +\beta_{1}\;{\text{Temp}}_{\mathrm{ijk}}+\beta_{2}\;{{\text{Temp}}_{\mathrm{ijk}}}^{2}\\ \;+\beta_{3}\;\log\left({\mathrm{VPD}}_{\mathrm{ijk}}\right)+\beta_{4}\;\log\left({\text{Soilm}}_{\mathrm{ijk}}\right)\\ \;+\beta_{5}\;\log\left({\mathrm{CI}}_{\mathrm{ijk}}\right)+a_{k}+a_{j|k}+\varepsilon_{\mathrm{ijk}}. \end{array}$$

Here, LUE_*ijk*_ is the measured LUE for 15‐day window *i*, of year *j*, at site *k*; *a*_*k*_ is a random intercept that allows for variation between sites; *a*_*j|k*_ is a random intercept that allows for inter‐annual variation at site *k*. The term *ε*_*ijk*_ is the residual (unexplained) error and includes intra‐annual variation not explained by the fixed factors.

**FIGURE 1:**
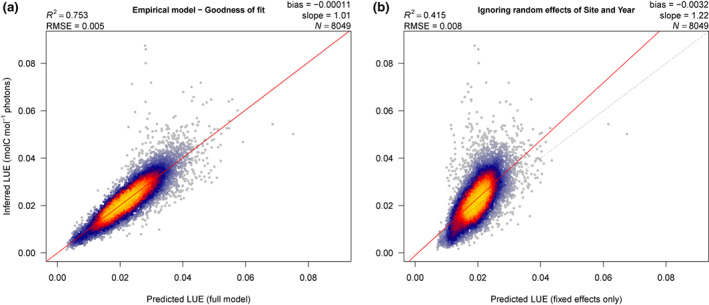
Goodness of fit between modeled and inferred light use efficiency; the model presented is M06 per Table 1. Each point represents one 15‐day average for a given site; the colored heat mapping provides an estimation of point density. The dashed line (in gray) shows the ideal 1: 1 fit. Panel (a) shows the fit for the full model while panel (b) shows the fit when variation in light use efficiency is explained using only the model's fixed term (i.e., the additive effects of temperature, vapor pressure deficit, soil moisture, and Cloudiness Index).

Predictions of LUE generated by the empirical model (M06) showed good agreement with the values inferred from eddy covariance data (Figure 1a). The scatterplot is evenly displaced around the 1:1 line, indicating that the model avoids systematic bias. Diagnostic plots raised no serious concerns (not shown). A further test predicting LUE based only on the fixed term of the statistical model (i.e., ignoring random effects, Figure 1b) showed weaker agreement, most notably fitted values were constrained to a narrower range than the inferred values. A leave‐one‐out cross‐validation of model performance (model iterations were trained using a dataset pruned of a single site, then tested using forcing data from that site; Figure S6) gauges how applicable the empirical model is to new sites not included in its development. In running the validation, we applied a generalized linear model with no random term since the full design (Equation 5) is unable to generate predictions for a site not included in the training set. The cross‐validation exercise therefore corresponds to Figure 1b that evaluates predictions generated by the empirical model's fixed term; a comparison of the two sets of predictions revealed very comparable performance (respective *R* ^2^ metrics of 0.39 and 0.41) and showed a similar tendency toward a narrower than observed range of LUE. We infer that site‐to‐site and year‐to‐year variations in LUE are not fully described by the fixed effects alone, but are successfully captured by the random effects of site and year in the full model.

### 3.2 Fixed effects

**FIGURE 2:**
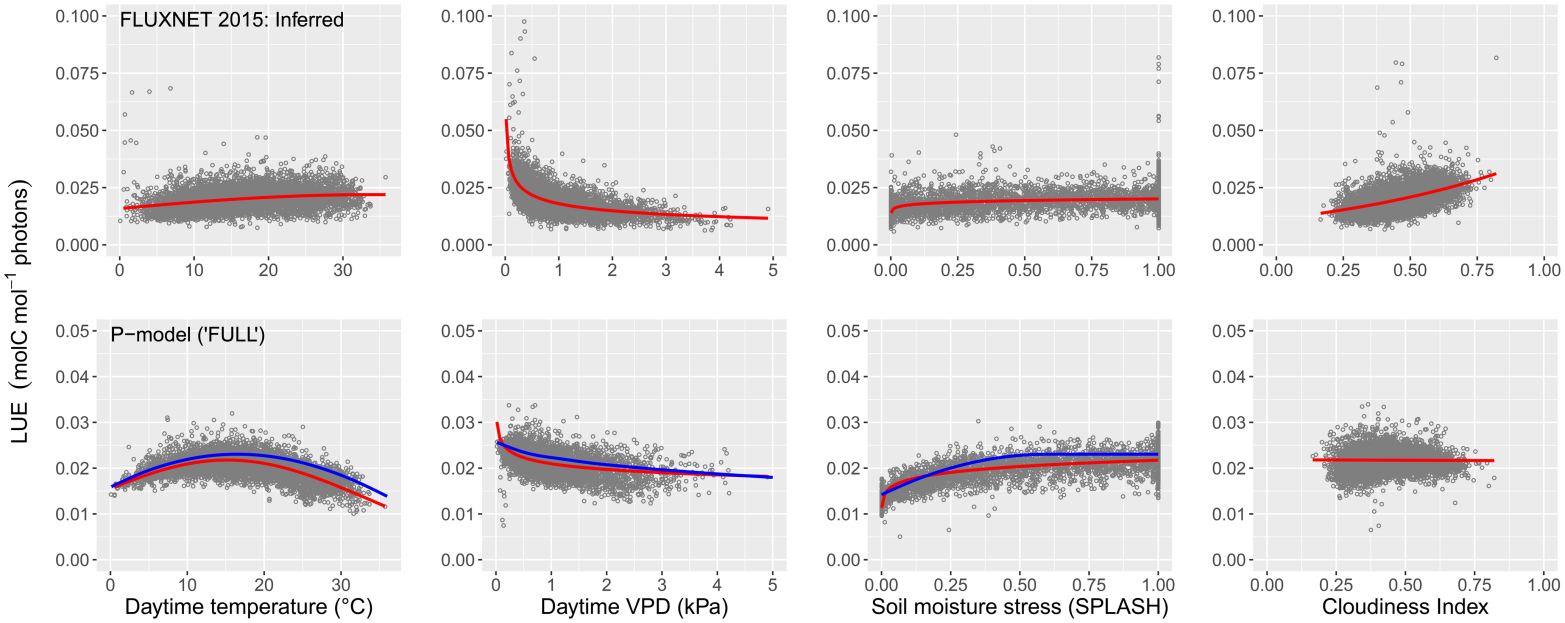
The dependencies of light use efficiency (LUE) on temperature, vapour pressure deficit, soil moisture, and Cloudiness Index. Top: conditional plots with partial residuals from our empirical model (M06, Table 1); estimated responses relate to inferred LUE values (FLUXNET). Bottom: conditional plots for the “FULL” implementation of the P model (Stocker et al., 2020) as applied to the main dataset; responses shown in red relate to LUE simulations. Overlaid in the bottom panel are LUE predictions (blue lines) generated by the P‐model for a dummy dataset that replicates the observed ranges of the environmental variables. The P‐model does not include an explicit term for diffuse radiation and so no dummy predictions are presented in the final panel. Notice that the response axes vary between the two rows. Each point represents a 15‐day average for a given site.

The nonlinearity of environmental relationships with LUE is apparent in Figure 2 (top panel) and reinforced by the marked inferiority of M08 in Table 1. There is a pronounced ranking of effects, with VPD >> diffuse fraction > temperature > soil moisture (see also Table S3). The temperature response appears to level off as daytime temperatures approach 30°C; the model coefficients (Table S3) imply a notional temperature optimum at 33.6°C, but the rarity in this dataset of 15‐day averages above 30°C means caution is required when interpreting responses at elevated temperatures. There is a strong inverse relationship between LUE and VPD, but a strong positive relationship with diffuse fraction. Modeled soil moisture effects are evidently important (compare M06 and M07 in Table 1), but the influence of soil moisture on LUE response is confined to a much narrower range than that of VPD.

### 3.3 Random effects and residuals

**FIGURE 3:**
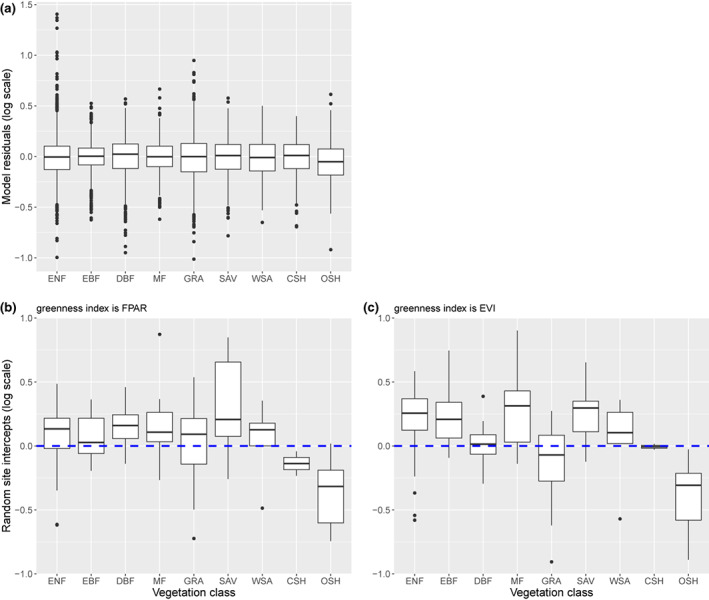
Diagnostic plots for the preferred empirical model (M06, Table 1) organized by vegetation class: (a) model residuals (15‐day averages); (b) random site intercepts corresponding to the main analysis and calculating fAPAR using the MODIS_FPAR index; (c) a comparative plot for a model run where light use efficiency (LUE) was calculated using the MODIS_EVI vegetation index. The box and whiskers in each case show the median result as a thick horizontal band. The ends of the box denote the interquartile range; the whiskers extend 1.5 times the interquartile range or to the most extreme value, whichever is smaller, and any points outside these values are shown as outliers. For all plots, the *y*‐axis scale represents log(LUE). For example in panel (b), the dashed horizontal line represents the population intercept of 0.010 mol C mol^−1^ photons (being exp^(−4.61)^ per Table S3); a random intercept term here of +0.13 (as per the median for ENF sites) corresponds (all model explanatory terms at zero) to an LUE of 0.011 mol C mol^−1^ photons (being exp^(−4.61+0.13)^).

Variation in LUE estimates *not* explained by the fixed term of the statistical model (i.e., the additive effects of temperature, VPD, soil moisture, and diffuse fraction) was apportioned to components of the random term as follows: inter‐annual 0.11; between sites 0.30; residual (including intra‐annual) variation 0.59. Inter‐annual variation, not captured by the climate inputs, could arise from plot disturbances invisible to remote sensing or changes in species composition. Figure 3 illustrates the model's residuals and random site intercepts, with sites categorized by vegetation type. There is no obvious pattern in the residuals (Figure 3a). The random site effects broadly overlap between most vegetation types. There is an indication, however, that shrublands tend to have lower than average LUE—albeit with fewer participating sites relative to forests (Table S1).

### 3.4 Comparison with the P‐model

In Figure 2 (bottom panel), LUE predictions generated with the P‐model using the same inputs from the observational dataset have been fitted using the empirical M06 (i.e., substituting predicted for inferred LUE as the response variable). Figure 2 also shows separate P‐model experiments, whereby predictions were generated by varying one explanatory term at a time (a dummy dataset provided a regular sequence for each of temperature, VPD, and soil moisture across their observed ranges) while the other drivers were held constant at their observed median values. No such experiment was possible for the cloudiness term since currently no provision is made in the P‐model for the effects of diffuse radiation—also explaining the lack of response evident in the conditional plot. Comparison of the fitted responses (red and blue lines) confirms that this statistical analysis of P‐model outputs has captured the nature of the P‐model's in‐built environmental responses.

There are notable differences between the P‐model's environmental responses and the effects on LUE seen in the data. In particular, the asymptotic relationship with temperature over the observed range in growth temperature is markedly different from the P‐model prediction of a temperature optimum at 15.4°C. Also, the observed reduction in LUE with increasing VPD is stronger than predicted.

### 3.5 Data‐model comparisons

**FIGURE 4:**
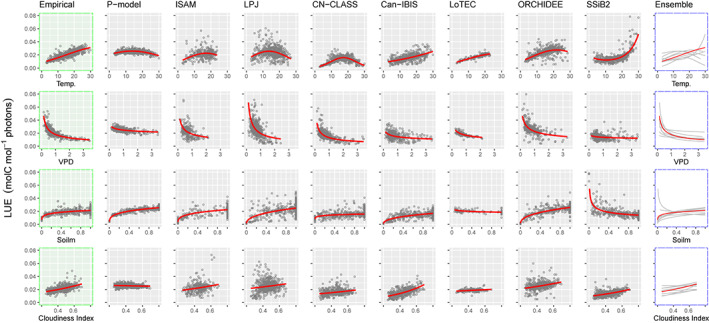
The relationships between environmental variables (air temperature, vapor pressure deficit, soil moisture, and Cloudiness Index) and light use efficiency predictions as generated by alternative models. Only seven of the participating NACP models are included here, see supplementary material for a companion plot (Figure S7). The first column is based on the empirical model as in Figure 2 (top panel). The second column is based on the FULL implementation of the P‐model (Stocker et al., 2020). The final column provides an ensemble figure of the varying responses with the empirical model indicated in red. Details of the NACP participating models are given in Table S2. Not all models generated gross primary production estimates for every site and time point.

We fitted the preferred empirical model (M06) to the portion of our global dataset that overlaps with the North American Carbon Program (NACP), thereby allowing use of the common site‐based flux data to compare the environmental dependencies of LUE as simulated by multiple models. The inter‐model comparison plots (Figure 4; Figure S7) illustrate that the strength, the shape, and even the sign of the estimated relationships vary substantially across the TBMs. Temperature effects are variously shown as peaked, nearly flat, or monotonically increasing. Inverse responses to VPD are shown in all cases, but with large differences in magnitude among models. Soil moisture effects are shown as increasing, flat, saturating, or declining while diffuse fraction effects range from flat to exponential. Direct comparisons with earlier figures (above) are not possible because we focus here on a much smaller subset of the data provided by the overlap of FLUXNET and NACP (12 sites only, as described earlier). In three cases (BEPS, CN‐CLASS, LoTEC), the common model design (Equation 5) as applied to simulated LUE generated convergence warnings, so some caution is required in interpreting those responses.

## 4 DISCUSSION

Several early empirical analyses of eddy covariance GPP data at a global scale have considered annual values and their dependence on macroclimatic variables such as mean annual temperature (MAT) and mean annual precipitation (MAP), or annual water‐balance indices. Based on data from 34 sites, Law et al. (2002) found that a combined index based on MAT and water balance explained 64% of variation in annual GPP. In a study of 513 forest sites, Luyssaert et al. (2007) found that power functions of MAT and MAP and their interaction explained 71% of variation in annual GPP. Exploiting the range of temporal data generated by the flux towers, later studies have considered variations in GPP at shorter time‐steps (e.g., Restrepo‐Coupe et al., 2013) and replaced annual means with more nuanced measures of environmental drivers (e.g., Fu et al., 2022). Our objective was to delve into the processes governing GPP, by factoring out the (otherwise dominating) influence of absorbed light; and considering predictor variables with a known, mechanistic connection to photosynthesis.

The LUE principle implies that GPP depends on light absorbed by green tissues, which is jointly determined by incident light and fAPAR. Incident light depends predictably on latitude, season, elevation, and cloud cover whereas fAPAR is strongly limited by water availability. This limitation is globally important for GPP (Nemani et al., 2003; Stocker et al., 2019; Wang et al., 2014). Ratios of assimilation to transpiration in C_3_ plants range from 2 to 11 mmol C mol^−1^ H_2_O (Lambers et al., 2008), emphasizing the high rate at which plants must lose water in order to fix carbon. This unfavorable exchange rate means that water availability limits the leaf area that can be displayed, and therefore the fAPAR (and GPP) that can be achieved, over much of the land surface.

Compared to the major effects of geographic variation in absorbed light and the limitation of fAPAR by water supply, the additional controls of GPP—that is, the controls of LUE as studied here—are relatively muted (Wang et al., 2014), but nonetheless significant. LUE is expected to be influenced by temperature because of the various temperature‐dependent quantities that influence photosynthetic rates (Bernacchi et al., 2001, 2003), and by VPD because of the universal response of stomata (Grossiord et al., 2020; Lin et al., 2015)—which progressively close to restrict transpiration, and therefore also photosynthesis, as VPD rises. Low soil moisture can be shown experimentally (Zhou et al., 2013) to increase the sensitivity of plants' stomata to VPD and, below a soil‐moisture threshold, also to reduce photosynthetic capacity. The diffuse radiation fraction influences the ability of incident light to penetrate the canopy and affects its distribution between sun and shade leaves.

The temperature response of leaf‐level photosynthesis is classically described by a parabola with photosynthesis inhibited (for different reasons) at low and high temperatures, reaching an optimum somewhere in between (Berry & Björkman, 1980). This, however, describes the instantaneous response and does not allow for acclimation of photosynthetic traits. There is debate on whether the facility for temperature acclimation differs between species (Yamori et al., 2014). However, most plants can adjust their photosynthetic characteristics to suit their growth temperature. Kumarathunge et al. (2019) showed that although the short‐term response of photosynthesis to temperature follows an optimum curve, with a steep decline at super‐optimal temperatures, an acclimation response systematically shifts the location of the optimum so that growth under higher temperatures results in a higher optimum, and therefore greater photosynthesis, than would have occurred otherwise. Geographic patterns of net primary production (GPP minus autotrophic respiration) also show a muted response to temperature (Michaletz et al., 2014). GPP temperature optima at canopy scale, inferred from eddy covariance data, peak at higher temperatures in warmer climates (Huang et al., 2017). These findings from large‐scale analyses are consistent with the expectation that acclimation might result in a less pronounced response of GPP to seasonal and spatial patterns of typical growth temperature than would be predicted based on the short‐term, experimentally manipulated response of photosynthesis. Consistent with this, our analysis suggests temperature optima increasing progressively with the time‐step considered: 14.8°C daily, 20.6°C weekly, and 33.6°C 15‐day (Figure S5). The apparent asymptotic relationship of LUE with temperature (Figure 2) is at odds with a recent study by Duffy et al. (2021) who reported a global thermal maximum for C_3_ photosynthesis of 18°C. Compared with our simple empirical modeling approach, the study by Duffy et al., although based on the FLUXNET2015 dataset, adopts a very different methodology with temperature responses defined by equations describing enzyme thermodynamics that rely on multiple coefficients likely to show temporal, spatial, and genetic variation.

As for the P‐model simulations, the approximately parabolic response of LUE to temperature (Figure 2) reflects the dependencies of two reactions that hinge on the dual affinity of the Rubisco enzyme and its relative specificity for the two substrates: CO_2_ (giving assimilation) and O_2_ (whereby carbon is lost by the plant through photorespiration). The kinetics of carboxylase–oxygenase are such that the quantum yield efficiency of photosynthesis declines with temperature while the CO_2_ compensation point increases. The latter (termed Γ* and a key parameter in the FvCB model) is the partial pressure of CO_2_ in the chloroplast at which photorespiratory CO_2_ loss equals the rate of carboxylation. This temperature dependence of Γ* is very well established (e.g., Bernacchi et al., 2001) and so it remains to be seen whether the inclusion of acclimation functions such as proposed by Kumarathunge et al. (2019) will alter the shape of the predicted LUE response curve or only shift the optimum along the temperature axis.

VPD influences CO_2_ uptake and water loss through progressive stomatal closure. Stomata respond within minutes to changes in VPD. This response is well described at the leaf level by models in which stomatal conductance declines with increasing VPD (Medlyn et al., 2011; Oren et al., 1999; Prentice et al., 2014). In addition, plants growing in warmer environments tend to maintain more open stomata and higher ratios of leaf‐internal to ambient CO_2_—as has been demonstrated using leaf‐level experiments on plants in different regions (Lin et al., 2015), global leaf stable carbon isotope data (Wang et al., 2017), and experimental manipulation of plant growth temperature (Marchin et al., 2016). The effect of VPD is also observed at canopy scale, as an important limitation on GPP (Lasslop et al., 2010; Zhang et al., 2019). We therefore expected, and found, a strong inverse relationship of LUE to VPD.

Soil moisture and VPD effects can be challenging to separate because low soil moisture is often accompanied by high VPD (e.g., Novick et al., 2016). Nonetheless, our data allowed sufficient independent variation in these two quantities to enable robust estimation of the responses of LUE to both variables. At the canopy scale, effects of low soil moisture on LUE, in addition to the effects of high VPD, can be observed in eddy covariance data from drier biomes (Stocker et al., 2018). Our results are consistent with the analysis of Stocker et al. (2018) showing a modest soil moisture effect that is steeper at low soil moisture values. A recent study by Fu et al. (2022), exploiting the European drought of 2018, found that when soils are wet, moderate drying could have a positive effect on GPP; and that the relative importance, for GPP, of VPD and soil moisture depends on the prevailing soil water conditions, with soil moisture dominating in the driest soils. Such findings appear to conflict with the indication by Liu et al. (2020) that soil moisture (rather than VPD) is the dominant control of GPP. This discrepancy remains to be resolved, but arguably the interaction between VPD and soil moisture is of more significance that their relative importance as individual terms.

The empirical positive relationship between LUE and diffuse fraction conformed with expectations. The inter‐model comparisons (Figure 4), however, generated a range of predicted effects from flat to exponential. In general, models simulating radiative transfer through the canopy, or that differentiate sun and shade leaves, have a mechanism to account for the diffuse effect; this is in contrast to “big‐leaf models” such as the P‐model.

Countering our expectation that the diffuse radiation effect would increase for denser canopies, we found no statistical evidence of an interaction with fAPAR. A general feature of ratio‐based indices, such as FPAR, is their asymptotic behavior, potentially leading to insensitivity to vegetation variations. In the case of dense canopies, the spectral reflectance algorithms saturate and are therefore weakly sensitive to changes in canopy properties. Finally, the diffuse fraction effect appears to show different patterns depending on time‐step—becoming shallower as we move from daily to weekly to 15‐day averages (Figure S5). That may chiefly be a product of the averaging step—with virtually no 15‐day CI averages >0.75.

We found no role in our preferred model for variations in ambient CO_2_ suggesting that the changes captured here were too small to affect canopy productivity.

We note below several potential sources of uncertainty in our analysis, which fall into two broad categories. On the one hand, the data we use might not provide us with what we imagine. On the other hand, we could be missing significant causal effects or interactions.

### 4.1 Data imprecisions

Inherent uncertainties in eddy covariance data have been comprehensively reviewed elsewhere (e.g., Baldocchi, 2003). The methodology has matured, however, and the FLUXNET data protocols (e.g., quality control and gap‐filling) inspire confidence (Pastorello et al., 2020). The best method for partitioning NEE continues to prompt debate, and concerns of possible systematic bias. For example, under the standard method (Reichstein et al., 2005), nocturnal *R*_eco_ is extrapolated to daytime conditions using temperature sensitivity estimates (so‐called nighttime partitioning). In a 3‐year study at Harvard Forest, Wehr et al. (2016) exploited the different carbon isotope signatures of photosynthesis and respiration to produce isotopic flux estimates. Their analysis indicated that *R*_eco_ was lower in the day than night—an effect ascribed to light inhibition of respiration (e.g., Sharp et al., 1984). The authors argued that, for the temperate forest they studied, nighttime partitioning methods overestimate *R*_eco_, and hence GPP, in the first half of the growing season. A global study by Keenan et al. (2019) reported evidence for light inhibition of respiration in a range of ecosystems, suggesting probable and pervasive overestimation of eddy covariance‐inferred estimates of GPP where night‐time partitioning is employed.

Remote imaging products, such as spectral vegetation indices (SVIs), have proved diagnostic of vegetation geography and phenology, allowing quantitative assessments of global vegetation state and change with the benefits of global coverage and frequent repetition. Difficulties remain, however. Time lags may occur between remotely sensed changes in greenness and photosynthetic activity. For temperate deciduous species, this decoupling might arise early or late in the growing season. An observed lag in GPP behind LAI during leaf emergence can be attributed to sustained investment in photosynthetic capacity beyond foliation (e.g., Barr et al., 2007). Conversely for evergreen needleleaf forests, photosynthetic recovery in spring may precede any detectable change in greenness, perhaps by as much as one month (Walther et al., 2016). Our threshold approach to data exclusion (Methods) should help to filter out false starts, and offsets between inferred and modeled LUE did not show any pronounced seasonal patterns (Figure S8). Apart from the challenges posed by dense canopies (above), surface reflectance algorithms might work better for some types of vegetation than others; particular challenges have been reported for boreal evergreen forests, where changes in greenness can be confounded or contaminated by snow cover (Walther et al., 2016). At sites with sparse vegetation, such as semi‐arid shrublands, SVIs may be affected by seasonal changes in solar elevation angle independent of the quantity of green vegetation (Sims et al., 2006). Substituting EVI for FPAR in our calculation of LUE had no effect on the structure of the preferred statistical model (Table S4). That substitution did, however, generate subtle differences in the pattern of site‐based random intercepts (Figure 3c) and that may be attributable to the lesser tendency perceived at forested sites for EVI values to saturate (see Figure S4). Lower apparent LUE for shrublands was a consistent finding.

Ideally, such an analysis would consider the temperature of the canopy, rather than the air. Disparities between canopy and air temperature are likely to be most pronounced under conditions of high temperature and water limitation when transpiration rates are reduced and leaf temperatures consequently elevated. Land surface temperature (LST) could provide a useful remotely sensed proxy for canopy conditions, provided LAI is sufficiently large. Sims et al. (2008) evaluated a GPP model driven only by EVI and LST at 11 flux sites in North America and found strong correlations between eddy covariance and modeled 16‐day estimates of GPP for selected forest sites, but not for a drought‐prone, shrubland site. Global application of LST as an indicator of canopy temperature will require methods to correct for the influence of bare ground in sparse vegetation types, and to estimate diurnal cycles of LST.

Soil moisture data present many difficulties (e.g., Vereecken et al., 2016). Here, for want of consistent, standardized ground measurements of soil moisture, we rely on simulations (e.g., Granier et al., 2007). We found only a modest correlation (*r* = 0.417) between simulated and observed soil moisture (not shown). However, only 14 sites in our dataset include measurements at a depth of 50 cm or greater. Remotely sensed measures of soil moisture, likewise, do not give information about the moisture content of deeper soil layers that can be essential for plant function (e.g., Matheny et al., 2017). Our simulation approach has the merit of being applied consistently across sites and considering the whole soil profile. Nonetheless, the algorithm is stylized. For example, it does not explicitly account for how vegetation properties, such as wilting point or rooting depth, influence evapotranspiration (Davis et al., 2017; Smith‐Martin et al., 2020). This must be noted as a caveat.

### 4.2 Missing processes

Our assumption of a linear dependence of canopy productivity on light is a simplification, adopted because it allowed us to factor out the otherwise dominant role of absorbed light in determining photosynthesis. A further iteration of our empirical model that included PPFD as an additional explanatory term did improve performance (lower AIC, BIC). The modeled effect was weakly negative such that LUE declined at higher irradiance (Figure S9), independent of the diffuse fraction. Assessments of the linear LUE assumption in the literature are somewhat contradictory. Mäkelä et al. (2008) developed an empirical model to predict variation in GPP at five European eddy covariance sites and found, as here, that temperature and VPD were important explanatory factors, but with no consistent role for SWC. For four of the five sites, an empirical nonlinear light parameter proved statistically significant and appeared positively related to latitude. For a tropical forest site, Ibrom et al. (2008) found that LUE systematically declined as a function of absorbed light although the variation in LUE was higher at daily than monthly timescales. Koyama and Kikuzawa (2010) found that for upper leaves of three temperate species, daily photosynthesis did not show light saturation, even under full light conditions. In our extended analysis, the PPFD term was much the weakest of the explanatory variables in the revised model (from the model summary, *F*‐value for soil moisture 165.8 vs. 50.7 for PPFD).

Many current TBMs, including half of the NACP ensemble (see table 2 in Schaefer et al., 2012), include explicit consideration of nitrogen (N) cycling and allow for an influence of N availability on leaf‐level photosynthesis. Photosynthesis correlates with leaf N due to the substantial investment of N in proteins and pigments that are directly involved (Evans, 1989). Earlier papers have provided evidence for “plant‐centered” control of leaf N and photosynthetic capacity (Dong et al., 2017; Smith et al., 2019). That is, it has been shown to first‐order, leaf‐level photosynthetic capacity (and, therefore, the photosynthetic component of leaf N) of unfertilized vegetation is optimized to the physical growth conditions (light, CO_2_, temperature) of the plant. This implies that the prime effect of restricted N supply is to reduce carbon allocation to leaves, as is commonly observed in N fertilization experiments. However, this approach is certainly a simplification and overlooks, for example, the influence of low soil phosphorus availability (Bloomfield et al., 2014; Peng et al., 2021) and soil pH (Paillassa et al., 2020) on photosynthetic capacity. Thus, differences in soil nutrient availability may have contributed to site‐specific variations in LUE that are captured by the random, rather than the fixed, term of our statistical model.

Most TBMs require parameter values to be estimated for each of a series of plant functional types (PFTs). Distinctions among PFTs can include genuine physiological or anatomical differences (e.g., C_3_ vs. C_4_ photosynthesis; angiosperms vs. gymnosperms), but often also include distinctions between plants from different climates. Many gas‐exchange studies have indeed found different behavior between PFTs or geographical subsets (Lin et al., 2015; e.g., Reichstein et al., 2007; Stocker et al., 2018; Turner et al., 2003). However, differences in fitted parameter values for plants growing in different climatic zones could simply reflect differences in growth environments. For example, estimated values of the average biome marginal water cost of carbon gain—ranging from 250 (mol mol^−1^) for cool conifer forests to 1500 for tropical seasonal forests, as reported by Lloyd and Farquhar (1994)—are consistent with the large differences in VPD between these environments. Differences in the stomatal sensitivity parameter *g*_1_, found by Lin et al. (2015) to correlate with the temperature in species' native ranges, can be explained by the effects of increasing temperature on photorespiration (increasing the cost of photosynthesis) and the viscosity of water (reducing the cost of water transport; Prentice et al., 2014). Our data analysis results are equivocal on the existence, or otherwise, of systematic differences in LUE among C_3_ PFTs. Site random effects grouped by vegetation type show no evidence for a systematic difference between, for example, ENF (gymnosperm‐dominated) and other forest types. Shrublands show a tendency to lower LUE. We cannot exclude the possibility that this tendency is a consequence of lower reliability of surface reflectance products applied to sites presenting sparse vegetation with a significant proportion of bare ground (e.g., Sims et al., 2006; Turner, Ritts, Cohen, et al., 2006).

### 4.3 Lessons from the multi‐model comparison

The design and relatedness of the models participating in the NACP have been discussed elsewhere (Huntzinger et al., 2013). Several TBMs participating in the project were here excluded as designed for agricultural settings, or unable to generate simulations for the sites intersecting with our dataset. In an evaluation of the GPP simulations using averaged, daily data from 39 flux sites, Schaefer et al. (2012) found that none of the 26 models matched measured GPP within the range of uncertainty of the observed fluxes. While the GPP models vary greatly in their complexity and representation of biological processes, the authors found that performance was independent of model structure or key characteristics. The models failed to show good, consistent agreement at any single site although performance was generally better for forests than grasslands or savannas—perhaps linked to overestimation of GPP under water‐limited conditions. In a supplementary exercise, we applied the Nash–Sutcliffe measure of model efficiency (Legates & McCabe, 1999): defined as the ratio of mean square error (the squared differences between observed and simulated values) to the variance in the observed data, subtracted from unity. The ratio ranges from minus infinity to 1.0 with higher values indicating better agreement. We found that none of the TBMs included here achieved a ratio greater than 0.4 (Table S5); that is, even for the best model, the MSE was 0.6 of the variance in the observed GPP values.

Our analysis goes further in revealing the dependencies between GPP and climate shown by the participating models. We expect to see a hump‐shaped response to temperature. Schaefer et al. (2012) reported an optimum of 20 (±5) °C (see their figure 8). But after controlling for covarying effects of VPD, soil moisture, and diffuse fraction, we find that simulations for some models (e.g., SSiB2, Can‐IBIS: Figure 4) show GPP increasing exponentially with temperature. That might be the result of a predominant role for water availability in those models or indicate the need for a better inhibition response in the upper temperatureranges. Photosynthetic thermal acclimation is common and observable over a period of weeks during the growing season (e.g., Berry & Björkman, 1980; Togashi et al., 2018), but such adjustments are ignored by many extant TBMs (LPJ and its successors constitute a known exception). It may also be important to consider respiratory acclimation in this context, since mitochondrial respiration can affect measurements of net photosynthetic rate even when photosynthesis is unaltered (Way & Yamori, 2014). Our analysis shows a consensus on the general nature of the response of GPP to changes in VPD, but the strength of the modeled inverse response varies greatly, and is almost negligible in some models. Consistent with our empirical analysis, soil moisture dependencies appear muted for most models. However, several depart from the anticipated positive trend over the transition between soil moisture limited and well‐watered conditions.

Although the NACP dataset was released 10 years ago, the disparity among TBMs revealed here, and the qualitative differences between observed and modeled dependencies on climate for many models and climate variables raise concern about model evaluation practices. The lack of realism in many models' inferred responses of GPP to individual climate variables would not have been detected by a typical benchmarking analysis based on goodness‐of‐fit metrics with flux measurements—in the original model‐data comparison, Schaefer et al. (2012) reported that the GPP simulations showed correlation coefficients between 0.6 and 0.9. Moreover, these responses presumably originate from incorrect process formulations that, at the time of model release, had not been adequately tested against relevant observations. The P‐model, which is substantially simpler and more transparent than most TBMs, shows qualitative agreement with our analysis of the GPP observations—certainly as they relate to VPD and soil moisture. Yet here too, we have demonstrated problems (temperature responses) or omissions (diffuse fraction) that were not noticed in the conventional data‐model comparison presented by Stocker et al. (2020), and that demand further investigation.

We conclude that developers should pay greater attention to the evaluation of specific process representations in models, to avoid incorrect environmental responses. We suggest that the typical “benchmarking” approach to model evaluation, although providing a necessary minimal test of model competence, is insufficient to ensure that models are not achieving the right results for the wrong reasons (e.g., by compensating erroneous process representations by varying parameter values unrealistically across PFTs). Assessing functional relationships, for example as incorporated in The International Land Model Benchmarking system (Collier et al., 2018), is a much‐needed addition to model development and evaluation. Finally, we note that the accumulation of publicly available flux data, along with remotely sensed vegetation measurements, has considerable potential to provide novel insights into the function of terrestrial ecosystems.

## CONFLICT OF INTEREST

The authors declare no competing interests.

## Supporting information


Figures S1

Tables S1

## Data Availability

The data that support the findings of this study are openly available on Zenodo at https://doi.org/10.5281/zenodo.7270055. These data were derived from the following resources available in the public domain: FLUXNET2015, https://fluxnet.org//data/fluxnet2015‐dataset/. NACP, https://daac.ornl.gov/NACP/guides/NACP_Site_Model_Flux_Std_Fmt.html. The scripts that implement the analysis are available on Zenodo at https://doi.org/10.5281/zenodo.7270260.
