# Supplementary material for: Environmental controls on the light use efficiency of terrestrial gross primary production
Source: Glob Chang Biol. 2022 Nov 25;29(4):1037–53. doi: 10.1111/gcb.16511 (PMC10099475; doi:10.1111/gcb.16511)
Supplement: Supplementary file 1 — Figures S1 [file GCB-29-1037-s002.docx]

# Environmental controls on the light use efficiency of terrestrial gross primary production

Keith J. Bloomfield, Benjamin D. Stocker, Trevor F. Keenan, I. Colin Prentice

Corresponding author: Keith Bloomfield (k.bloomfield@imperial.ac.uk)

## Supplementary information, figures

# Supplementary figures

| Figure S1 | Site locations, a global map |
| --- | --- |
| Figure S2 | The relationship between diffuse and total transmission |
| Figure S3 | Time-series of FPAR estimates; selected sites |
| Figure S4 | Choice of vegetation greenness index |
| Figure S5 | Choice of timescale: daily, weekly or 15-day |
| Figure S6 | Goodness of fit plot under cross validation (companion plot to Figure 1, main text) |
| Figure S7 | Functional relationships for NACP TBMs (companion plot to Figure 4, main text) |
| Figure S8 | Seasonal patterns in inferred and modelled LUE |
| Figure S9 | Conditional plot of PPFD effects on LUE |

Fig S 1 Global map indicating those 117 FLUXNET sites included in the analysis. Notice the poor representation for the tropical zone.


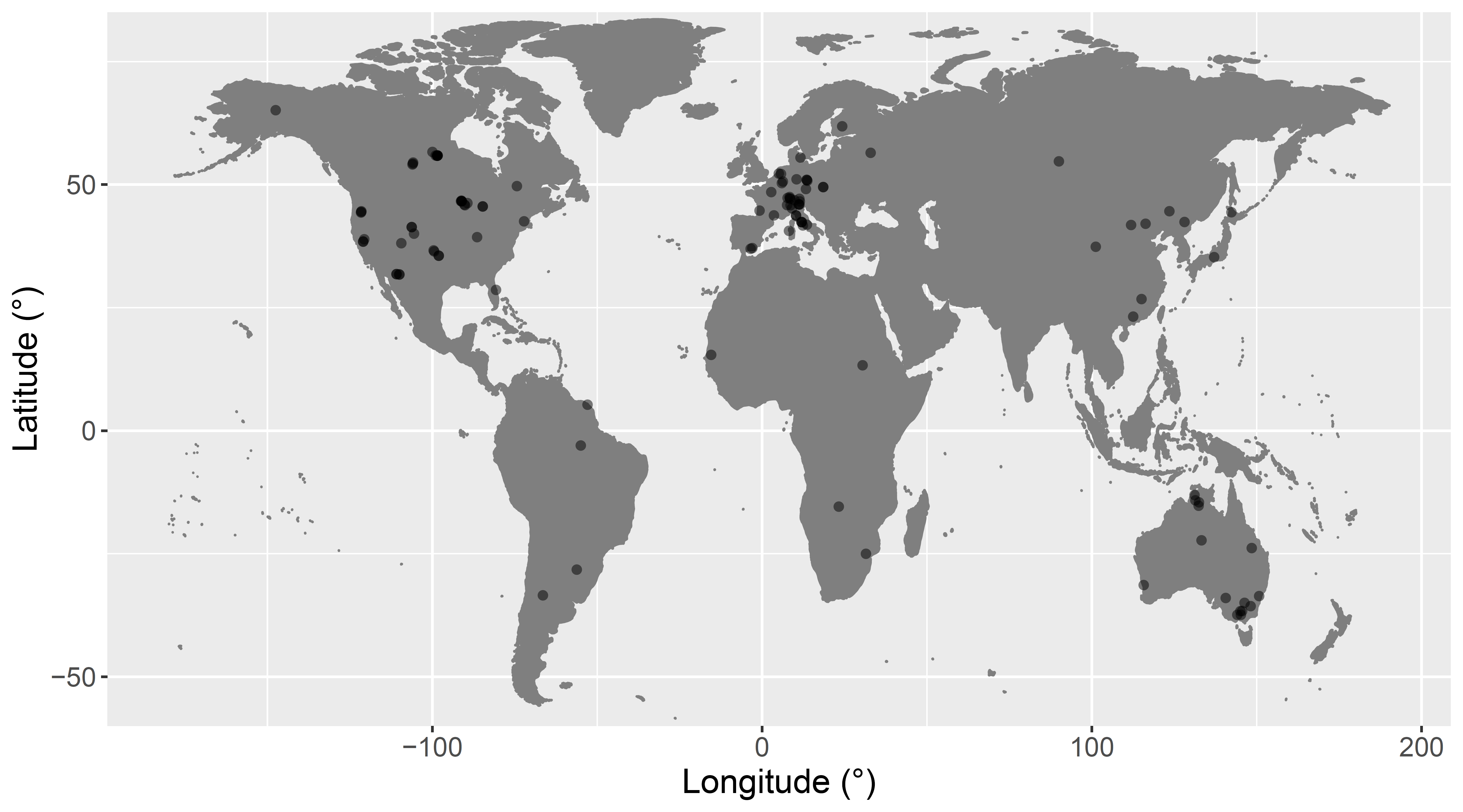


Fig S 2 Diffuse transmittance as a function of total transmittance after Bristow et al. (1985).


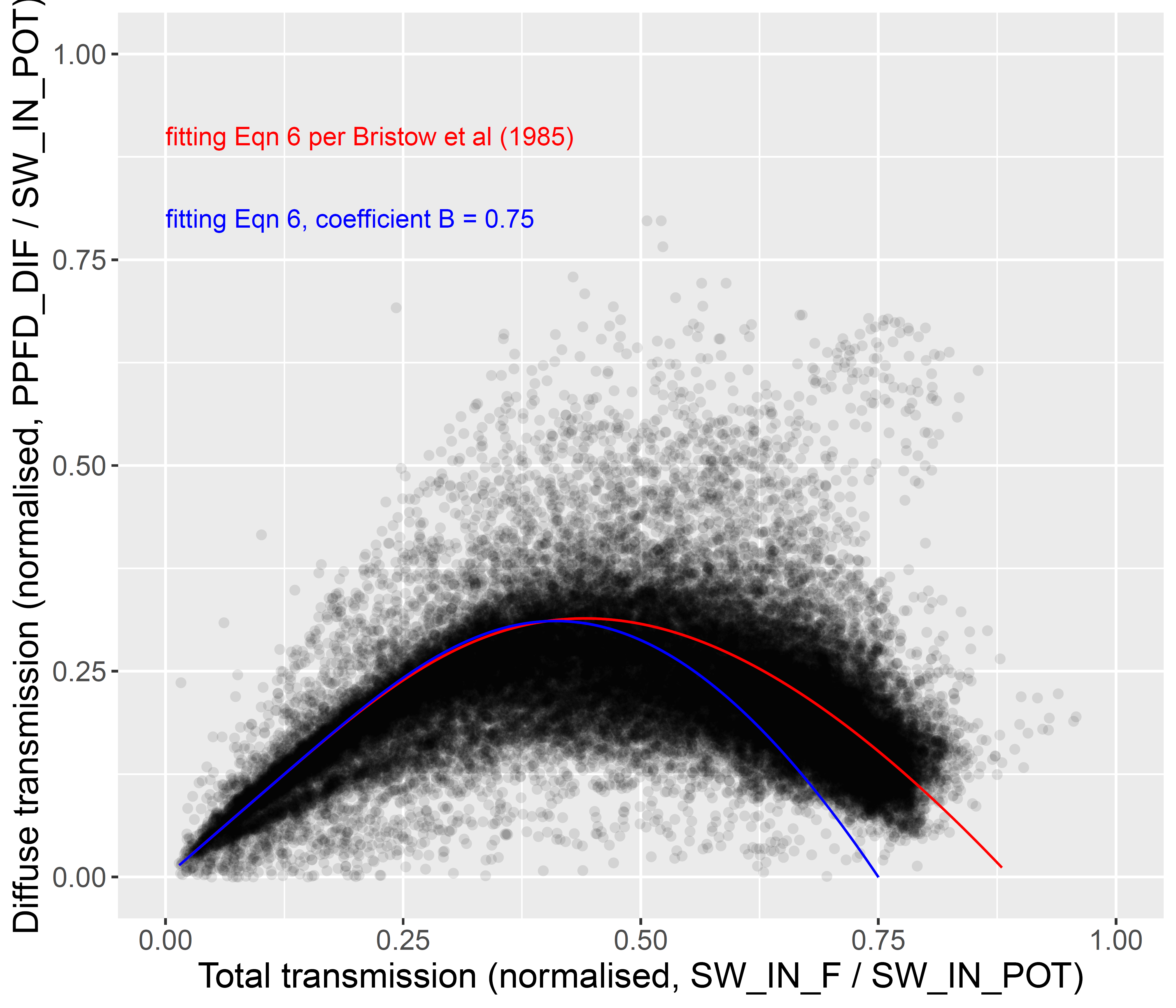


Figure S 3 Timeseries of gap-filled FPAR indices for selected sites (Table S1 for details), chosen as representative of the major vegetation classes in our dataset.


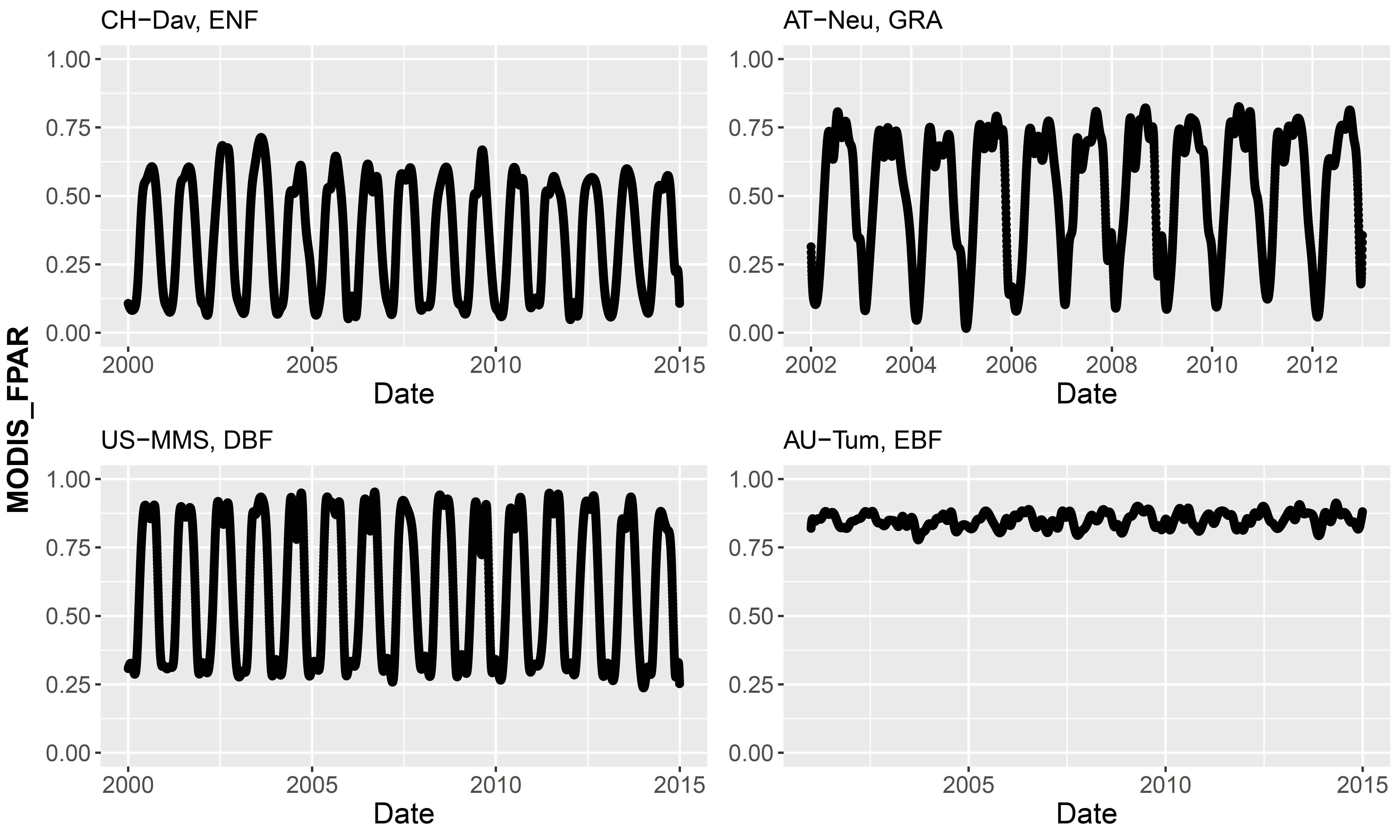


Fig S 4 Scatterplots, organised by vegetation category (IGBP classification), of the correlation between two remote-sensing estimates of vegetation conditions: FPAR and EVI both generated by MODIS. Each point represents a 15-day average. The dashed red lines indicate the ideal 1:1 fit. Notice the tendency, especially pronounced in the forested sites, for FPAR values to saturate in the upper ranges (e.g. for EBF, an FPAR of 0.9 can correspond to a broad range of EVI values).


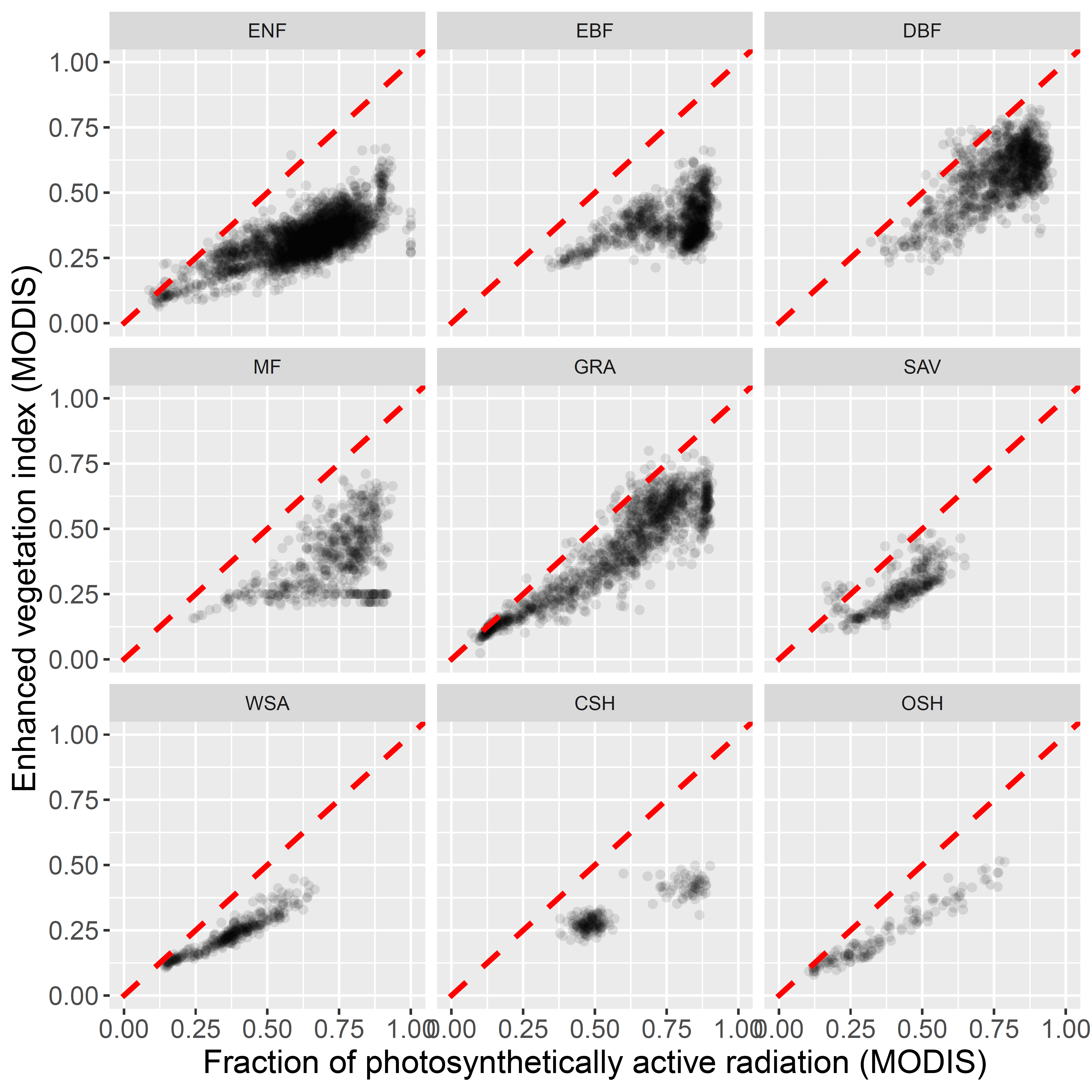


Fig S 5 The environmental dependencies of LUE simulations generated by the preferred empirical model design (M06, Table 1 main text), but using data averaged at a range of time-steps: daily, weekly and 15-day (as in the main analysis). The points are partial residuals. The driving variables are: air temperature (°C), vapour pressure deficit (kPa), soil moisture index and cloudiness index. The LUE relationship with air temperature is modelled here as a quadratic function, consistent with Equation (5) main text.


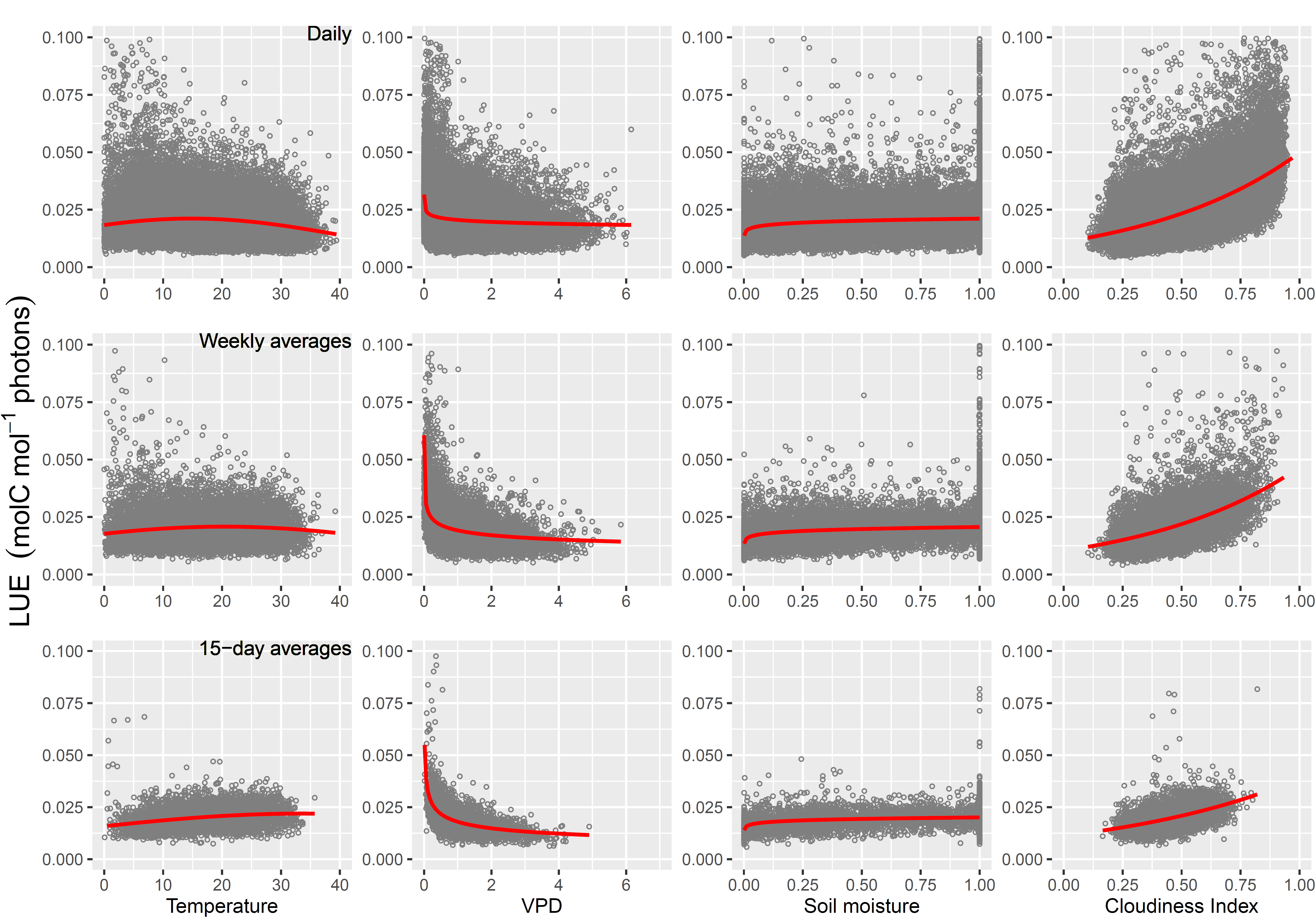


Fig S 6 Goodness of fit plot for inferred *versus* predicted LUE under a cross-validation exercise - model iterations were trained using a dataset pruned of a single site, then tested using forcing data from that site. Each point represents one 15-day average for a given site; the coloured heat mapping provides an estimation of point density.


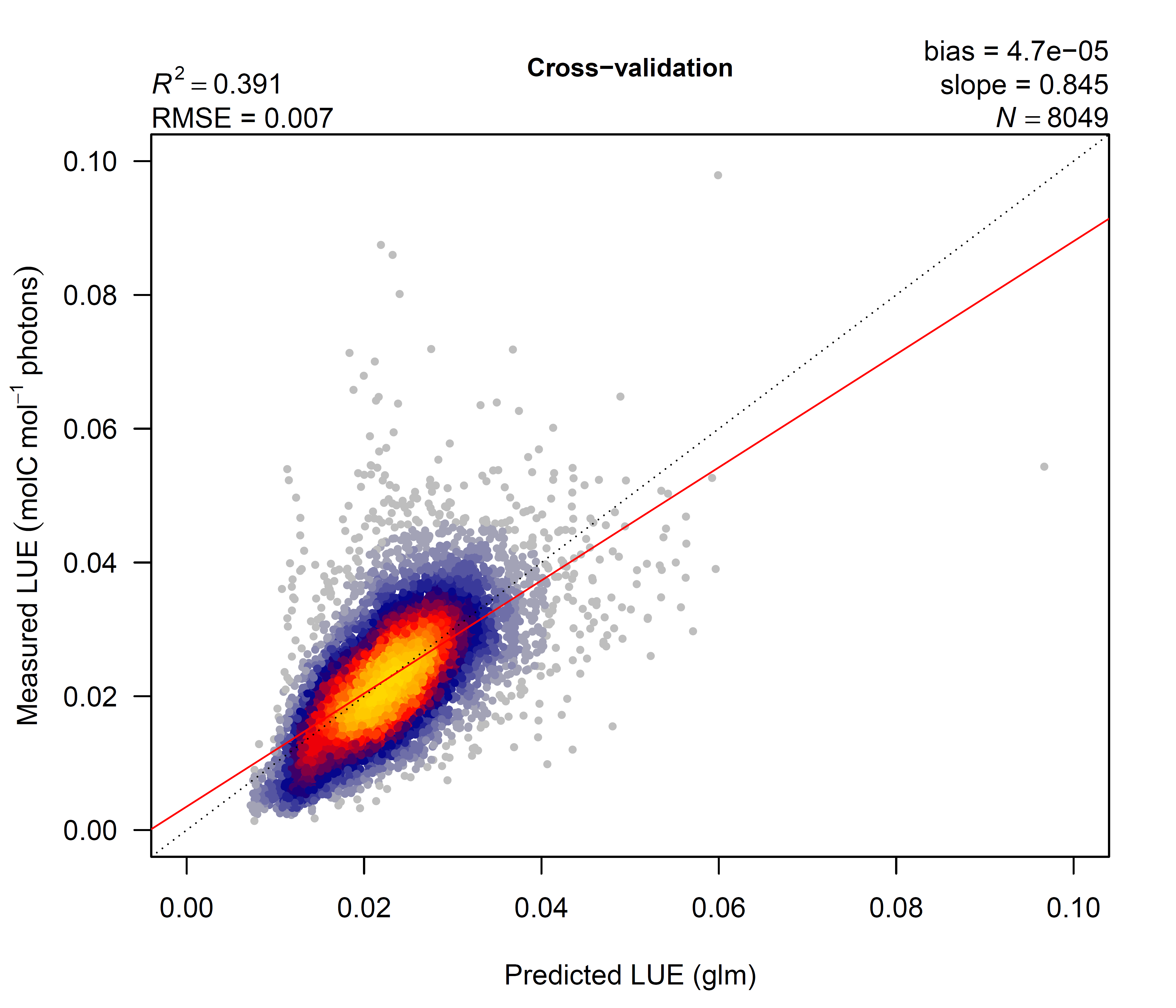


Fig S 7 Functional relationships with LUE as estimated by Terrestrial Biosphere Models participating in the North American Carbon Program. The final column provides an ensemble plot of the varying responses across the TBMs. Not all models generated GPP estimates for every site and time point. Companion plot to Figure 4 in the main text. Details of the participating models are provided in Supplementary [Table S 2](#Ref66440100).


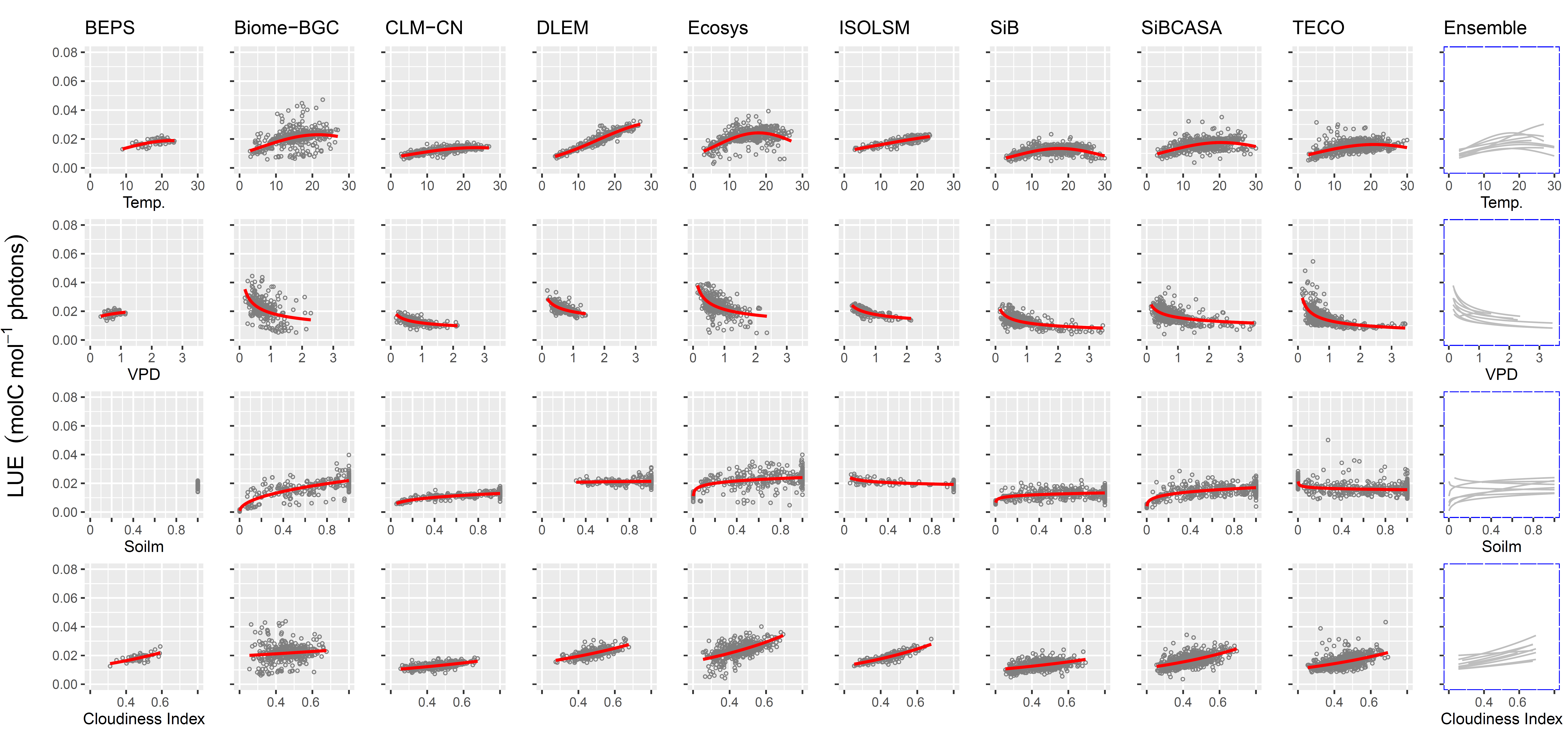


Fig S 8 Seasonal patterns to potential bias (modelled minus inferred) in LUE ratios. Points are based on 15-day averages.


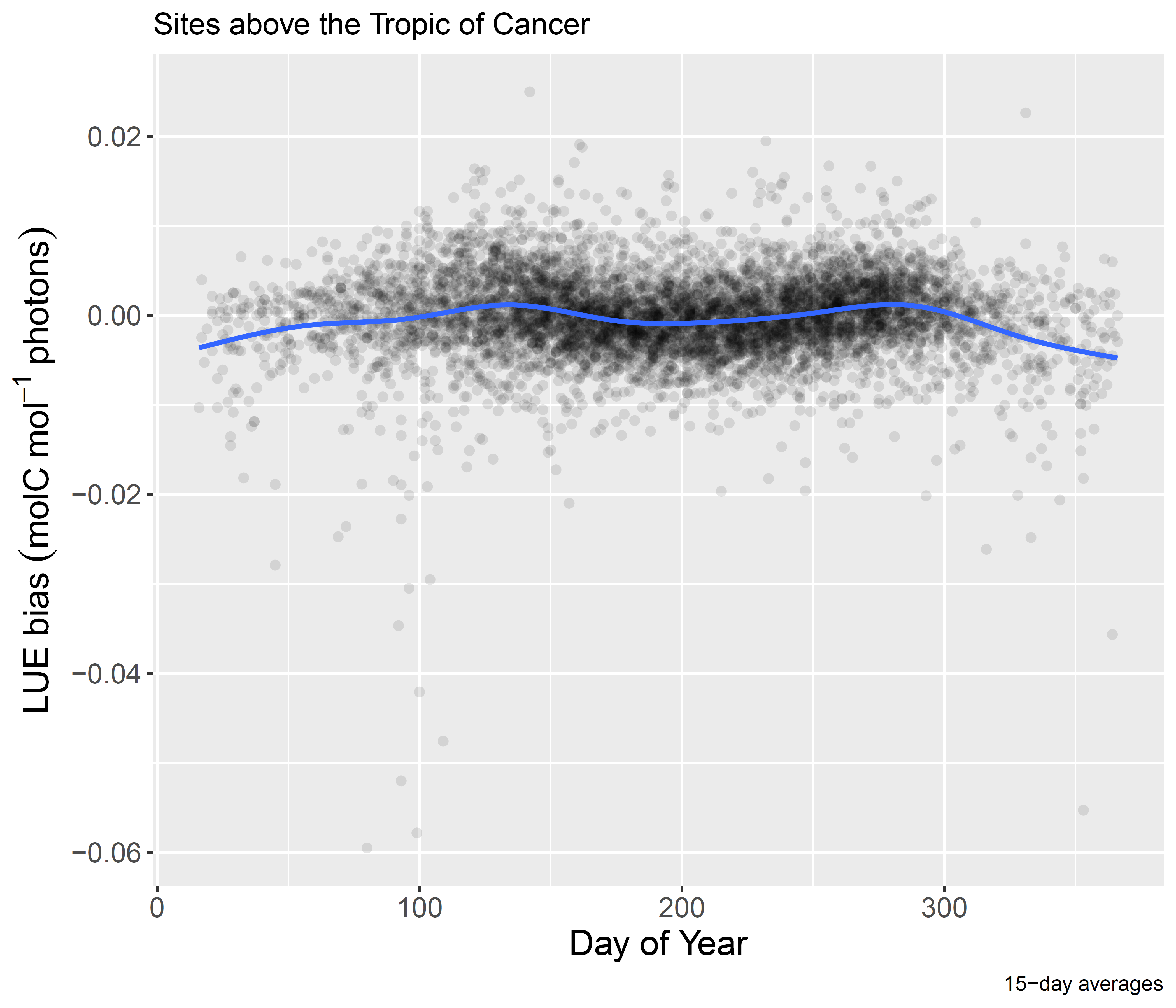


Fig S 9 Conditional plot showing the predicted effects of variation in light (PPFD) on Light Use Efficiency after controlling for the additive effects of temperature, VPD, soil moisture and diffuse fraction of solar radiation (i.e. other model terms held at median value).


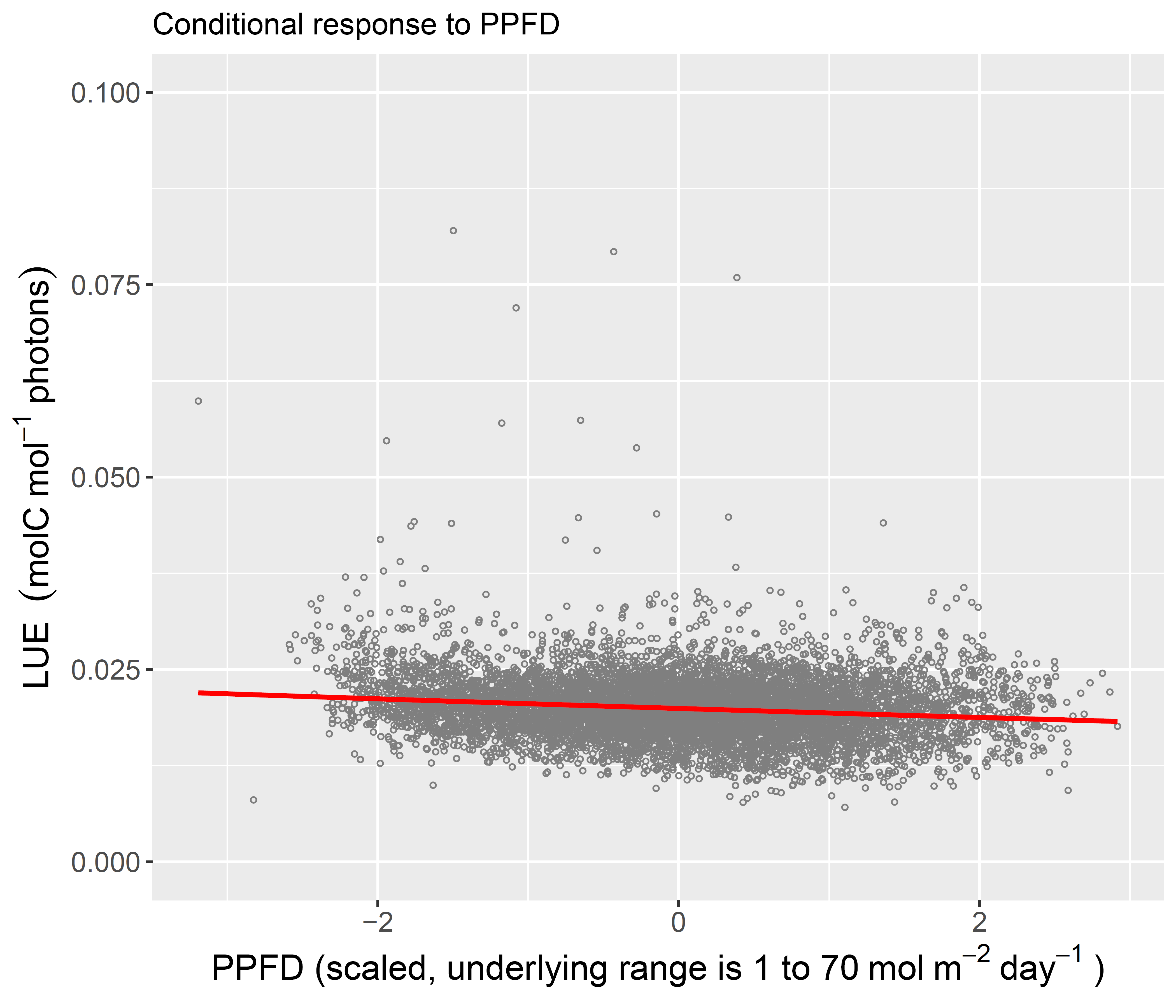


Bristow, K. L., Campbell, G. S., & Saxton, K. E. (1985). An equation for separating daily solar irradiation into direct and diffuse components. *Agricultural and Forest Meteorology, 35*(1-4), 123-131. doi:10.1016/0168-1923(85)90079-6
