# Supplementary material for: Environmental controls on the light use efficiency of terrestrial gross primary production
Source: Glob Chang Biol. 2022 Nov 25;29(4):1037–53. doi: 10.1111/gcb.16511 (PMC10099475; doi:10.1111/gcb.16511)
Supplement: Supplementary file 2 — Tables S1 [file GCB-29-1037-s001.docx]

# Environmental controls on the light use efficiency of terrestrial gross primary production

Keith J. Bloomfield, Benjamin D. Stocker, Trevor F. Keenan, I. Colin Prentice

Corresponding author: Keith Bloomfield (k.bloomfield@imperial.ac.uk)

## Supplementary information, tables

# Supplementary information

| Table S1 | FLUXNET site details |
| --- | --- |
| Table S2 | Participating models, North American Carbon Program |
| Table S3 | Coefficients for the empirical LUE model |
| Table S4 | Model selection steps (companion to Table 1, main text) with LUE derived using MODIS_EVI |
| Table S5 | NACP models: performance criteria |

Table S 1 Site details: longitude (negative values are west of the prime meridian); latitude (north as positive); vegetation categories follow the IGBP land cover classification: evergreen needleleaf forest (ENF), evergreen broadleaf forest (EBF), deciduous broadleaf forest (DBF), mixed forest (MF), closed shrublands (CSH), open shrublands (OSH), woody savannahs (WSA), savannahs (SAV) and grasslands (GRA); climate zone follows the Köppen climate classification; n refers to the number of discrete 15-day periods included in the main analysis (growing season); those sites that provided an overlap with the North American Carbon Program are also indicated.


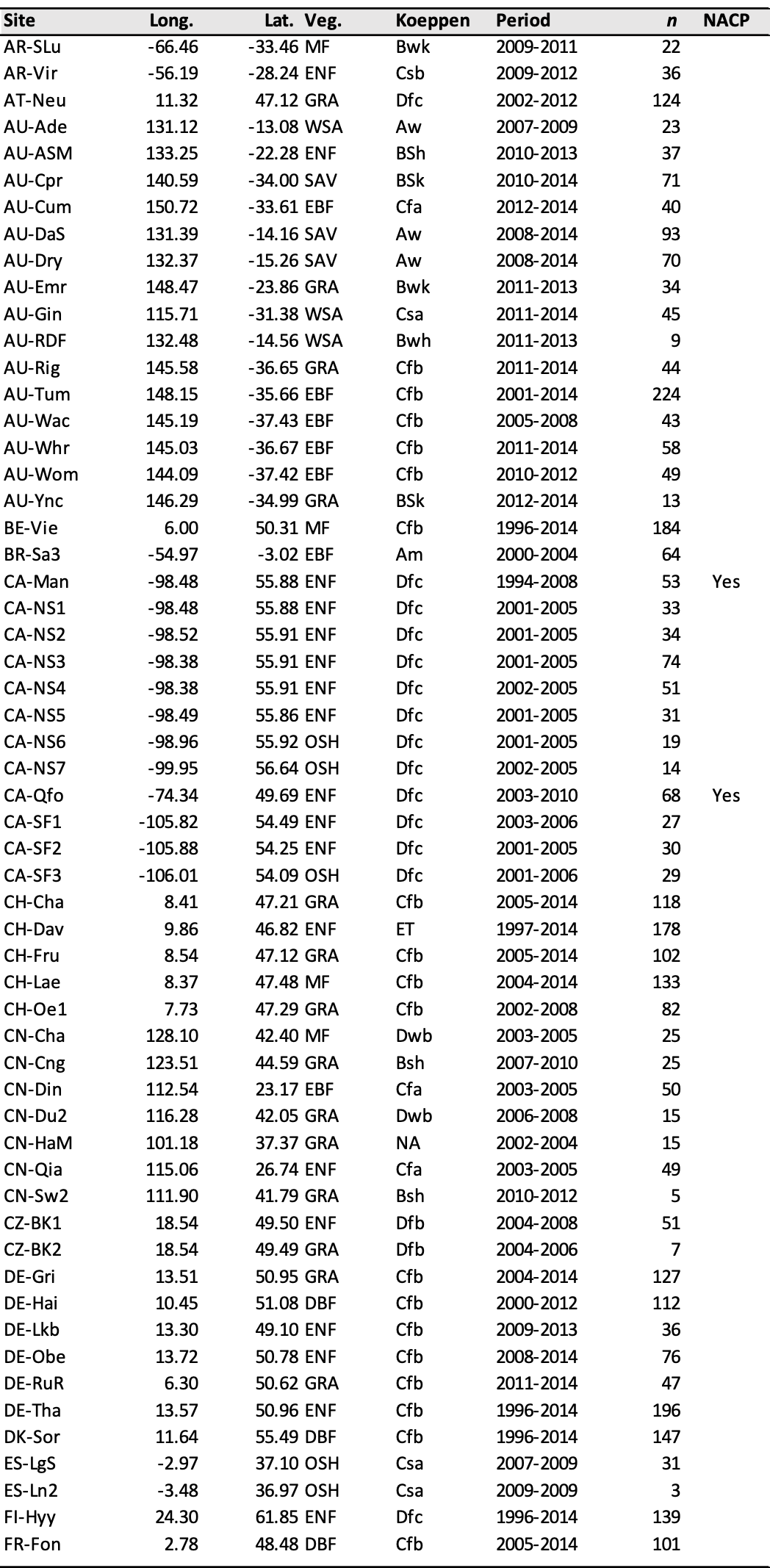


Table S1 *continued*


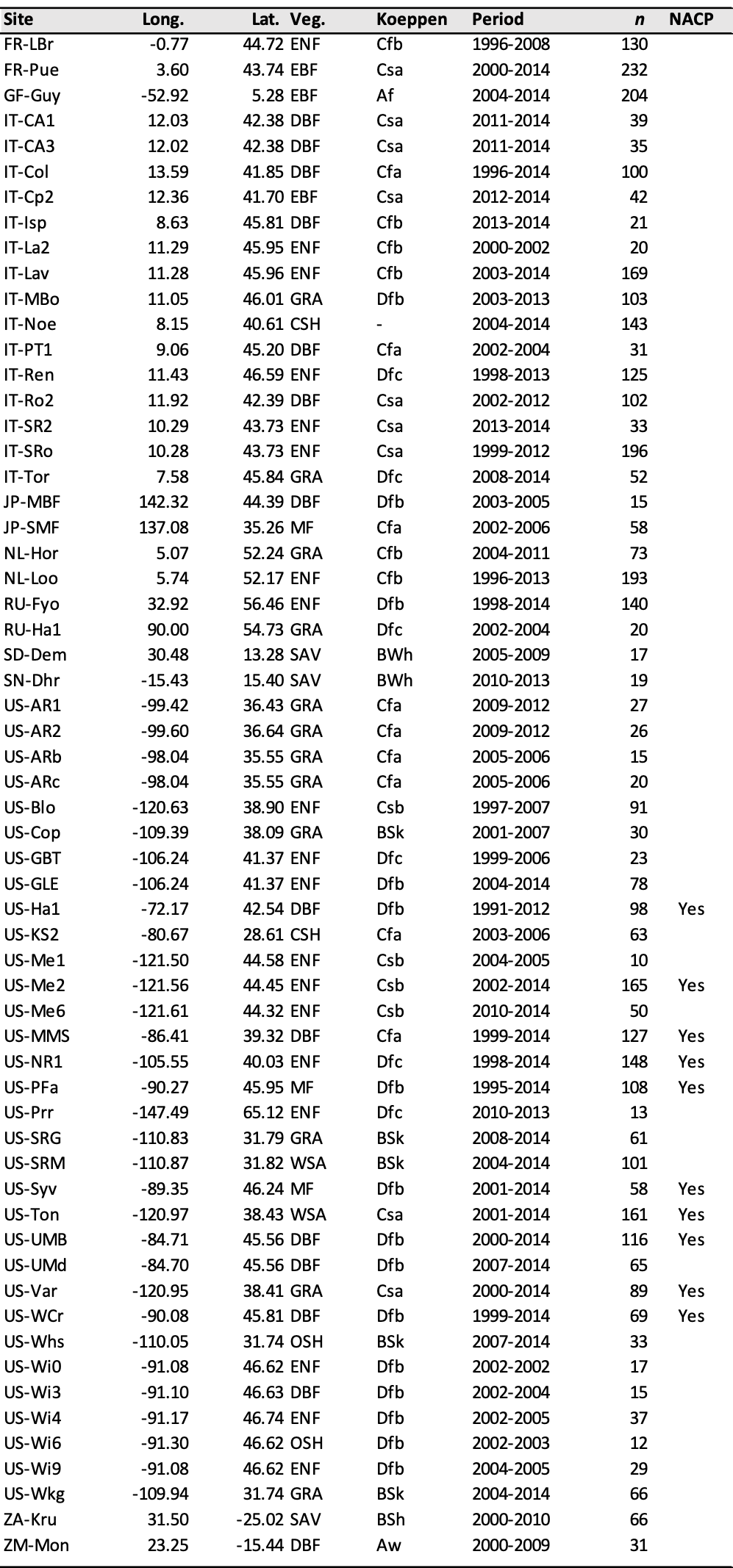


Table S 2 Participating terrestrial biosphere models for the North American Carbon Program providing estimates of GPP and LAI for site-day combinations that overlap with the main dataset. The twelve sites from the main analysis that overlap with the NACP dataset are indicated on [Table S 1](#Ref71720738). GPP model types: enzyme kinetic *versus* light use efficiency.

| **TBM** | **Number site-years** | **GPP model** |
| --- | --- | --- |
| BEPS | 7 | EK |
| Biome-BGC | 52 | EK |
| Can-IBIS | 58 | EK |
| CLM-CN | 35 |  |
| CN-CLASS | 76 | EK |
| DLEM | 39 | EK |
| Ecosys | 57 | EK |
| ISAM | 38 | LUE |
| ISOLSM | 20 | EK |
| LoTEC | 16 | EK |
| LPJ | 69 | EK |
| ORCHIDEE | 74 | EK |
| SiB3 | 76 | EK |
| SiBCASA | 76 | EK |
| SSiB2 | 77 | EK |
| TECO | 77 | EK |

Table S 3 Coefficients for the empirical model’s fixed term (Equation 5). The final model is a generalized linear mixed model with a log link term: hence the estimated intercept (all other variables being zero) corresponds to a light use efficiency of 0.010 mol C mol^-1^ photons. The coefficients are presented in two formats (a) main analysis: adopting orthogonal polynomials: 1^st^ and 2^nd^ degree (b) adopting raw polynomials for the temperature term (convergence warnings suggested possible numerical estimation problems). For a meaningful interpretation of the fitted model, we can use the raw coefficients per the worked example below:

1. Orthogonal polynomials:

1. Raw polynomials:

## Worked example:

Adopting typical mid-range values for the drivers: temperature 15 °C, vapour pressure deficit 0.8 kPa, soil moisture stress term 0.25 and Cloudiness Index of 0.5:

$$LUE= e^{\left\{ -4.786 + \left( 0.0184 *15 \right) + \left( {-0.0003}^{2} * 15 \right) + \left( -0.2686 * log\left( 0.8 \right) \right) + \left( 0.0531 * log\left( 0.25 \right) \right)+ \left( 1.2610 * 0.5 \right) \right\}}=0.021 mol {mol}^{-1}$$

$$T_{optimum}= {-0.0184}/{\left( 2 * -0.0003 \right)=33.65 ℃}$$

Table S 4 Model selection steps (companion to Table 1, main text); the response variable is LUE but with fAPAR estimates provided by MODIS_EVI rather than MODIS_FPAR. All models shared a common random design as per Equation (5). Candidate terms: daytime temperature (Temp), daytime vapour pressure deficit (VPD), soil moisture (Sm), elevation (Elv), ambient CO_2_, Cloudiness Index (CI) and fAPAR. df, degrees of freedom; AIC, Akaike Information Criterion; BIC, Bayesian Information Criterion; logLik, log likelihood.

Table S5 performance criteria for the NACP inter-model comparisons (model details per Table S2). The tests consider the fit between observed and predicted values of GPP: Coefficient of determination (*R*^2^), Root Mean Square Error (RMSE), Bias. The nsEC metric refers to the Nash-Sutcliffe efficiency criterion (Legates & McCabe, 1999): defined as the ratio of MSE to the variance in the observed data, subtracted from unity. The models are ranked in descending order of nsEC.

Legates, D. R., & McCabe, G. J. (1999). Evaluating the use of "goodness-of-fit" measures in hydrologic and hydroclimatic model validation. *Water Resources Research, 35*(1), 233-241. doi:10.1029/1998wr900018
